# Exploiting two-dimensional morphology of molybdenum oxycarbide to enable efficient catalytic dry reforming of methane

**DOI:** 10.1038/s41467-020-18721-0

**Published:** 2020-10-02

**Authors:** Alexey Kurlov, Evgeniya B. Deeva, Paula M. Abdala, Dmitry Lebedev, Athanasia Tsoukalou, Aleix Comas-Vives, Alexey Fedorov, Christoph R. Müller

**Affiliations:** 1 Department of Mechanical and Process Engineering, ETH Zürich, Leonhardstrasse 21, CH 8092 Zürich, Switzerland; 2 Department of Chemistry and Applied Biosciences, ETH Zürich, Vladimir-Prelog-Weg 1-5, CH 8093 Zürich, Switzerland; 3 Department of Chemistry, Universitat Autònoma de Barcelona, 08193 Cerdanyola del Vallès, Catalonia Spain; 4 Present Address: Department of Materials Science and Engineering, Northwestern University, Evanston, IL 60208 USA

**Keywords:** Chemical engineering, Natural gas, Two-dimensional materials

## Abstract

The two-dimensional morphology of molybdenum oxycarbide (2D-Mo_2_CO_*x*_) nanosheets dispersed on silica is found vital for imparting high stability and catalytic activity in the dry reforming of methane. Here we report that owing to the maximized metal utilization, the specific activity of 2D-Mo_2_CO_*x*_/SiO_2_ exceeds that of other Mo_2_C catalysts by ca. 3 orders of magnitude. 2D-Mo_2_CO_*x*_ is activated by CO_2_, yielding a surface oxygen coverage that is optimal for its catalytic performance and a Mo oxidation state of ca. +4. According to ab initio calculations, the DRM proceeds on Mo sites of the oxycarbide nanosheet with an oxygen coverage of 0.67 monolayer. Methane activation is the rate-limiting step, while the activation of CO_2_ and the C–O coupling to form CO are low energy steps. The deactivation of 2D-Mo_2_CO_*x*_/SiO_2_ under DRM conditions can be avoided by tuning the contact time, thereby preventing unfavourable oxygen surface coverages.

## Abstract (web summary)

The two-dimensional morphology of molybdenum oxycarbide (2D-Mo_2_CO_*x*_) nanosheets dispersed on silica is found vital for the dry reforming of methane. Here the authors show that the specific activity of 2D-Mo_2_CO_*x*_/SiO_2_ exceeds that of other Mo_2_C-based catalysts by ca. 3 orders of magnitude.

## Introduction

Since the early work by Sinfelt^1^ and Boudart^2^ demonstrated that Mo_2_C and WC exhibit platinum-like catalytic activity in alkane hydrogenolysis and isomerization, a multitude of reports has appeared aiming to exploit molybdenum carbide for industrially-relevant reactions such as methane aromatization^3,4^, the water-gas shift^5,6^, and CO_2_ hydrogenation reactions^7,8^, to name just a few^9^. In contrast to middle-to-late transition metal-based catalysts that deactivate by the deposition of coke, sintering or poisoning^10^, carbide catalysts typically feature a low rate of coking^11^, are resistant to high temperature sintering as well as to sulfur poisoning^12,13^. However, the high oxophilicity of carbides fosters their evolution in reactions with oxygenate substrates, leading ultimately to deactivation via the formation of oxide phases; deactivated catalysts require regeneration by re-carburization with CH_4_/H_2_^11^. If such deactivation pathways are avoided while the metal utilization of carbides is maximized, for instance by developing catalysts with a high surface-to-bulk ratio and a high dispersion on supports, the replacement of traditional, more expensive middle-to-late transition metal catalysts by carbide-based catalysts will become viable.

The dry reforming of methane, (DRM, CH_4_ + CO_2_ ↔ 2CO + 2H_2_) is an example of a particularly challenging reaction for Mo_2_C-based catalysts because it combines a CO_2_-rich feed with high operating temperatures (typically, 800 °C and above)^11,14–21^. Deactivation of Mo_2_C in DRM conditions proceeds oxidatively according to Mo_2_C + 5 CO_2_ → 2 MoO_2_ + 6 CO^11,17,18^ and in order to mitigate it, dry reforming of methane is often conducted at elevated pressures (2–10 bar)^11,14,16,18,21^. From a mechanistic standpoint, an ill-defined molybdenum oxycarbide phase (MoO_*x*_C_*y*_) has been reported to play a key role in DRM^22^, as well as in the water-gas shift^23^, deoxygenation^24,25^, and CO_2_-to-methanol^8^ conversion processes, suggesting that the partial oxidation of Mo_2_C is favorable for those reactions. In addition, recent reports on CO_2_-propane dehydrogenation catalyzed by Mo_2_C highlighted that control over the oxygen coverage of the surface oxycarbide layer allows tuning the catalytic activity and selectivity^26,27^. Molybdenum oxycarbide species were also proposed as intermediates during the dehydroaromatization of methane on molybdenum-loaded zeolite ZSM-5^28^. However, establishing the local structure and the nature of the active sites in such molybdenum oxycarbide phases has remained challenging, largely because well-defined oxycarbide catalysts, serving as references, are not available.

In this context, a recently discovered family of two-dimensional atomically-thin early transition metal carbides, nitrides, and carbonitrides called MXenes^29^ could be exploited as an entry point to supported well-defined 2D carbides and oxycarbides. MXene films are nanocrystalline yet form stable colloidal solutions in protic solvents, and feature metal-terminated surfaces (vide infra) with a lateral size in the submicron scale, which yields highly uniform surface metal sites^30^. The atomically thin nature of MXenes enables optimal metal utilization provided that these films are highly dispersed on a support. The 2D morphology is also advantageous for mechanistic studies since bulk metal sites in catalysts derived from MXenes, are minimized. Therefore, the characterization methods applied (even when not being inherently surface sensitive) will provide information mostly about the surface sites, which is instructive for structure-activity studies.

In what follows, we describe an approach to yield a supported two-dimensional (2D) model molybdenum oxycarbide material containing well-defined surface sites that are highly active in the dry reforming of methane. We prepare this catalyst from colloidal, delaminated thin films of the molybdenum carbide MXene phase, Mo_2_C*T*_*x*_, where *T* stands for OH, O, F surface termination groups. After supporting Mo_2_C*T*_*x*_ on SiO_2_, these surface groups can be controllably removed (de-functionalized) by reduction in H_2_, giving 2D-Mo_2_C/SiO_2_. The oxidation of 2D-Mo_2_C/SiO_2_ by CO_2_ at 800 °C gives a 2D molybdenum oxycarbide 2D-Mo_2_CO_*x*_/SiO_2_ with a high oxygen surface coverage and an average Mo oxidation state of +5.5. Subjecting this material to DRM conditions reduces the oxygen coverage and forms a catalyst in situ with an average Mo oxidation state of +4. This material is highly active in DRM, in contrast to fully reduced 2D-Mo_2_C/SiO_2_ and 2D-Mo_2_CO_*x*_/SiO_2_ material with highest oxygen coverage (confirmed by a Mo oxidation state of ca. +5) which are essentially inactive for the DRM. Our Mo *K*-edge X-ray absorption near edge structure (XANES) and X-ray photoelectron spectroscopy (XPS) results verify that molybdenum oxycarbide is the active phase in Mo_2_C-based DRM catalysts^31^ and density functional theory (DFT) calculations identify that an oxygen coverage of ca. 0.7 monolayer provides a high activity of 2D-Mo_2_CO_*x*_/SiO_2_ in DRM. Reducing or increasing this optimal oxygen coverage decreases the reaction rate. 2D-Mo_2_CO_*x*_/SiO_2_ catalyst with reduced oxygen coverage is also less selective owing to a competing reverse water-gas shift reaction. Hence, supported 2D-Mo_2_CO_*x*_ catalyst with an initially high coverage of surface oxygen atoms activates first by reduction, that is the oxygen coverage is lowered until a maximum in DRM performance is reached at a Mo oxidation state of ca. +4. Reducing the Mo oxidation state further (i.e., lowering the oxygen coverage below ca. 0.7 monolayers) leads to catalyst deactivation. This observation is in sharp contrast to non-layered Mo_2_C-based catalysts that readily deactivate by oxidation in a CO_2_ atmosphere. It is therefore remarkable that deactivated 2D-Mo_2_CO_*x*_/SiO_2_ catalysts do not contain any deposited coke according to temperature-programmed oxidation (TPO) experiments and can be regenerated by oxidation in CO_2_, fully recovering their highest catalytic activity. Noteworthy, the deactivation of 2D-Mo_2_CO_*x*_/SiO_2_ under DRM conditions can be avoided by optimizing the contact time. Density functional theory calculations corroborate experimental observations and demonstrate that the DRM pathway on an oxycarbidic surface with a submonolayer oxygen coverage is energetically favored compared to the pathway occurring on a fully carbidic surface. The fully oxygen-covered surface is unstable in DRM conditions and reduces in situ its oxygen coverage below 1 oxygen monolayer, also consistent with our experiments.

## Results

### Synthesis and characterization

**Fig. 1:**
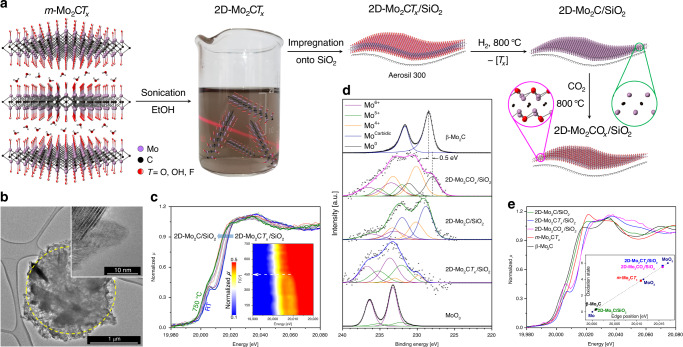
Synthesis and characterization of 2D-Mo_2_CO_*x*_/SiO_2_. **a** Synthesis of 2D-Mo_2_CO_x_/SiO_2_ from delaminated Mo_2_C*T*_*x*_ films via incipient wetness impregnation of their colloidal solution onto silica, followed by the reduction of the *T*_*x*_ groups under H_2_ (giving 2D-Mo_2_C/SiO_2_) and the subsequent oxidation in CO_2_ (giving 2D-Mo_2_CO_*x*_/SiO_2_); **b** TEM images of 2D-Mo_2_C*T*_x_/SiO_2_; **c** in situ reduction of 2D-Mo_2_C*T*_*x*_/SiO_2_ under H_2_ as followed by Mo *K*-edge XANES revealing an evolution of the pre-edge feature with temperature (shown in the inset as a contour plot); **d** oxidation state of Mo in reference and synthesized materials determined from the deconvolution of ex situ XPS data of the Mo 3*d* core levels as well as from **e** the edge position in XANES spectra.

Multi-layered Mo_2_C*T*_*x*_^32^ (referred to as *m*-Mo_2_C*T*_*x*_) was sonicated in ethanol to yield, after centrifugation, a transparent purple colloidal solution of delaminated 2D-Mo_2_C*T*_*x*_ flakes (Fig. 1a and Supplementary Fig. 2)^33^. A dried aliquot of this solution was analyzed by transmission electron microscopy (TEM) revealing single and a few-layer thin flakes of Mo_2_C*T*_*x*_, the latter morphology is identified by the characteristic scrolling of edges of the few-layer thin MXene nanosheets (Supplementary Fig. 3)^34^. The X-ray powder diffraction pattern of the dried delaminated material is typical of a layered Mo_2_C*T*_*x*_ structure (Supplementary Fig. 4)^35^. The colloidal solution of the Mo_2_C*T*_*x*_ nanoflakes (ca. 0.15 mg mL^−1^ concentration determined by thermogravimetric analysis) was used for several consecutive incipient wetness impregnations of a SiO_2_ support (Aerosil 300, calcined at 950 °C, 194 m^2^ g^−1^ surface area according to nitrogen physisorption), giving 2D-Mo_2_C*T*_*x*_/SiO_2_ that, after drying at 100 °C in air, contained 0.48 wt% Mo by elemental analysis. TEM imaging of 2D-Mo_2_C*T*_*x*_/SiO_2_ shows agglomerated amorphous silica grains that are homogeneously covered with thin Mo_2_C*T*_*x*_ nanosheets (Fig. 1b and Supplementary Fig. 5). Consistent with an MXene structure, hexagonally ordered Mo atoms are observed by (high resolution) HR-TEM in 2D-Mo_2_C*T*_*x*_/SiO_2_ (Supplementary Fig. 5). The X-ray powder diffraction (XRD) pattern of 2D-Mo_2_C*T*_*x*_/SiO_2_ reveals only an amorphous halo corresponding to SiO_2_ (Supplementary Fig. 6). The absence of crystalline peaks of *m*-Mo_2_C*T*_*x*_ confirms a high dispersion of individual delaminated Mo_2_C*T*_*x*_ nanosheets on the support.

Next, we fitted the experimentally acquired Fourier-transformed Mo *K*-edge extended X-ray absorption fine structure (EXAFS) functions of 2D-Mo_2_C*T*_*x*_/SiO_2_ with two coordination spheres, i.e., Mo−C/O/OH and Mo−Mo. The fitting of the first coordination sphere of Mo gives two scattering paths with interatomic distances of 1.74(1) and 2.03(1) Å and coordination numbers (CN) of 1.0(2) and 1.2(3), respectively (Table 1, Supplementary Fig. 12). The shorter scattering path is attributed to molybdenum oxo (Mo=O) surface sites since the fitted distance 1.74(1) Å is in good agreement with the reported Mo=O bond lengths of ca. 1.7 Å found in a molybdenum oxycarbide in a zeolite structure^31^ as well as in molecular Mo complexes^36,37^ (determined by EXAFS and X-Ray crystallography, respectively). The second scattering path of the first coordination shell with an interatomic distance of 2.03(1) Å is attributed to the Mo−C and Mo−O bonds where O belongs to a hydroxy surface termination site and C is the interlayer carbidic carbon, consistent with the 2.1 Å Mo−C distance in the parent *m*-Mo_2_C*T*_*x*_^32^. The second Mo coordination sphere is fitted with three Mo–Mo scattering paths with interatomic distances of 2.63(1), 2.92(2), and 3.42(2) Å and with coordination numbers of 0.8(4), 1.4(5), and 1.1(4), respectively (Table 1, Supplementary Fig. 12). The presence of three different Mo–Mo paths indicates a strong distortion of Mo sites in silica-supported Mo_2_C*T*_*x*_, deviating notably from the Mo coordination environment in parent *m*-Mo_2_C*T*_*x*_ that features only two Mo–Mo paths at 2.9 Å and 3.2 Å with coordination numbers of 4 and 2, respectively (as determined by EXAFS)^32^. This deviation is likely owing to (i) the higher fraction of Mo=O sites in 2D-Mo_2_C*T*_*x*_/SiO_2_ relative to the *m*-Mo_2_C*T*_*x*_, (ii) a disorder of 2D-Mo_2_C*T*_*x*_/SiO_2_ caused by the geometrical curvature of the delaminated nanosheets supported on the silica grains and (iii) the interaction with the support. All these factors could contribute to the lower total CNs of Mo in 2D-Mo_2_C*T*_*x*_/SiO_2_ as determined by EXAFS.

**Table 1:** EXAFS fitting of 2D-Mo_2_C*T*_*x*_/SiO_2_ (R factor 0.008).

Shell	CN	R (Å)	σ^2^ (Å^2^)	ΔE (eV)
Mo−O_1_	1.0 (2)	1.74 (1)	0.005 (2)	6 (2)
Mo−C/O_2_	1.2 (3)	2.03 (1)		
Mo−Mo_1_	0.8 (4)	2.63 (1)	0.006 (2)	
Mo−Mo_2_	1.4 (5)	2.92 (2)		
Mo−Mo_3_	1.1 (4)	3.42 (2)		

Subsequently, we explored the transformation of these silica-supported 2D-Mo_2_C*T*_*x*_ sheets in reductive, oxidative, and DRM conditions. Note that, unless specified otherwise, all these treated materials were characterized by avoiding exposure to air, using in situ experimentation, gloveboxes and air-tight transfers for characterization (XPS, fourier-transform infrared (FTIR) spectroscopy, TEM and XANES). Reductive de-functionalization of 2D-Mo_2_C*T*_*x*_/SiO_2_ from the passivating *T*_*x*_ surface groups was first studied in H_2_ flow by in situ XANES (Mo *K*-edge, Fig. 1c). Interestingly, in contrast to the bulk Mo_2_C*T*_*x*_^32^, a well-defined pre-edge feature, typical for molybdenum in a non-centrosymmetric environment^38,39^, appears in the room temperature XANES spectrum of 2D-Mo_2_C*T*_*x*_/SiO_2_ (Supplementary Fig. 13). Under H_2_ flow and with increasing temperature (in situ XANES experiment), the Mo *K*-edge in 2D-Mo_2_C*T*_*x*_/SiO_2_ shifts to lower energies and this process continues up to ca. 450 °C (from 20015.8 to 20002.0 eV), indicating a gradual reduction of Mo due to the de-functionalization of the *T*_*x*_ groups (Fig. 1c). Simultaneously, the intensity of the characteristic pre-edge feature of 2D-Mo_2_C*T*_*x*_/SiO_2_ reduces until its disappearance at ca. 450 °C. Increasing the temperature further to 750 °C induces only a minor shift of the Mo *K*-edge position compared to 450 °C, that is from 20002.0 to 20000.8 eV, the latter value is very close to the edge position of β-Mo_2_C (20000.7 eV). This suggests that H_2_ treatment at 750 °C leads to a Mo terminated carbidic surface predominantly free from oxo, hydroxy and fluoro terminations, supporting the assignment of the aforementioned pre-edge feature to molybdenum sites in 2D-Mo_2_C*T*_*x*_/SiO_2_ as opposed to MoO_2_ or MoO_3_ (conceivable oxidation products of 2D-Mo_2_C*T*_*x*_). Noteworthy, the temperature for the de-functionalization of most of the *T*_*x*_ groups in 2D-Mo_2_C*T*_*x*_/SiO_2_ (according to XANES) is approximately 450 °C, which is 150 °C lower than that of *m*-Mo_2_C*T*_*x*_^32^, probably owing to the high dispersion of the 2D-Mo_2_C*T*_*x*_ sheets on the silica surface that eliminates gas diffusion limitations in the pores of *m*-Mo_2_C*T*_*x*_.

Since the DRM reaction typically requires high temperatures to yield high equilibrium conversions (>700 °C)^40^, 2D-Mo_2_C*T*_*x*_/SiO_2_ was treated in a H_2_ flow at 800 °C for 1 h to obtain 2D-Mo_2_C/SiO_2_. This high temperature, reductive treatment did not affect significantly the original morphology and high dispersion of the silica-supported nanosheets, as confirmed by TEM imaging (Supplementary Fig. 8). That said, in few cases small nanoparticles (ca. 2–3 nm) decorating molybdenum carbide sheets could be discerned in TEM images (Supplementary Fig. 8) possibly due to the partial sintering of 2D-Mo_2_C sheets to Mo_2_C nanoparticles at areas where nanosheets overlap. Overall, 2D-Mo_2_C*T*_*x*_/SiO_2_ behaves very differently in reductive de-functionalization conditions when compared to *m*-Mo_2_C*T*_*x*_ that sinters under these conditions forming a porous bulk β-Mo_2_C phase^32^. Similarly, a multi-layer *m*-V_2_C*T*_*x*_ was reported to sinter around 600 °C, which appears to be a general temperature limitation of multi-layer MXenes^41^. These results underline the importance of the dispersion of the nanosheets on a support to avoid sintering. Next, we prepared silica-supported model 2D molybdenum oxycarbide 2D-Mo_2_CO_*x*_/SiO_2_ by treating 2D-Mo_2_C/SiO_2_ in a flow of CO_2_ (800 °C, 1 h, Fig. 1a). According to TEM, the CO_2_ treatment of 2D-Mo_2_C/SiO_2_ does not alter the morphology or dispersion of the supported nanosheets (Supplementary Fig. 9).

A comparison of the Mo *3d* XPS spectra of the prepared silica-supported materials and β-Mo_2_C and MoO_3_ references is given in Fig. 1d. The Mo *3d* spectrum of 2D-Mo_2_C*T*_*x*_/SiO_2_ was fitted with Mo^4+^, Mo^5+^, and Mo^6+^ components with the latter two being the dominating oxidation states (Supplementary Table 2). In contrast, the Mo *3d* spectrum of *m*-Mo_2_C*T*_*x*_ can be fitted using only Mo^4+^ and Mo^5+^ components (Supplementary Table 2)^32^ indicating that Mo sites are more oxidized in the silica-supported delaminated Mo_2_C*T*_*x*_ nanosheets than in *m*-Mo_2_C*T*_*x*_. In contrast, the Mo *3d* XPS spectrum of 2D-Mo_2_C/SiO_2_ is described by mostly carbidic Mo sites in addition to a smaller fraction of Mo^4+^ and Mo^6+^ sites (Supplementary Table 2). To understand better the nature of the Mo^4+^ and Mo^6+^ sites, observed by XPS of the reduced materials, 2D-Mo_2_C/SiO_2_ was analyzed by transmission IR spectroscopy revealing a low intensity band at ca. 2290 cm^–1^ that is tentatively assigned to surface [≡Si–H] sites (Supplementary Fig. 15). We speculate that the high temperature H_2_ treatment of 2D-Mo_2_C*T*_*x*_/SiO_2_ may give molybdenum hydrides (carbidic or oxycarbidic) that further react by opening the siloxane bridges of silica forming [Mo–O_s_] and [≡Si–H] bonds (Supplementary Fig. 15, inset). Such reactivity has been observed previously for surface hydrides of tantalum that cleaved siloxane bridges of silica^42^. Therefore Mo^4+^ sites in 2D-Mo_2_C/SiO_2_ are likely formed due to the grafting^43^ of the reduced nanosheets onto the silica surface.

Interestingly, the fitting of the *3d* XPS spectrum of Mo in 2D-Mo_2_CO_*x*_/SiO_2_ requires 4 components, namely Mo^6+^, Mo^5+^, Mo^4+^ and metallic Mo^0^ (Supplementary Table 2). Note that Mo^0^ is not observed in the XPS spectra of 2D-Mo_2_C/SiO_2_ and 2D-Mo_2_C*T*_*x*_/SiO_2_. We rationalize this result by a partial removal of the carbidic carbon from the 2D-Mo_2_C layer by CO_2_ at 800 °C (forming 2 equiv of CO) and attribute the presence of the Mo^6+^ component in the fit to the oxygen-rich oxycarbide phase of 2D-Mo_2_CO_*x*_/SiO_2_. In contrast, heating multi-layer *m*-Mo_2_C*T*_*x*_ in a CO_2_ flow transforms it into a mixture of β-Mo_2_C and MoO_2_ already at ca. 550 °C, according to our in situ XRD experiment (Supplementary Fig. 16). This result underlines again that dispersion and isolation of MXene nanosheets on a support is a critical step, prior to high temperature treatments in both reducing and oxidizing conditions, to retain the nanosheet morphology of the (oxy)carbide phase. In a sharp contrast to the stability of 2D-Mo_2_CO_*x*_/SiO_2_ in an atmosphere of pure CO_2_ at 800 °C, other supported Mo_2_C-based catalysts yield MoO_2_ already in CO_2_-rich DRM feeds, which is the main deactivation pathway of these catalysts^11,19^.

Next, ex situ Mo *K*-edge XANES spectra of 2D-Mo_2_C*T*_*x*_/SiO_2_, 2D-Mo_2_C/SiO_2_ and 2D-Mo_2_CO_*x*_/SiO_2_ were compared (Fig. 1e). Note that the edge position is defined as the first inflection point of the XANES spectra after the pre-edge feature (where the pre-edge corresponds to the forbidden *1s*-*4d* transition)^39^. In agreement with XPS results, 2D-Mo_2_C*T*_*x*_/SiO_2_ features an edge position at 20015.8 eV that is notably higher than in *m*-Mo_2_C*T*_*x*_ (20010.9 eV) indicating more oxidized Mo sites in supported Mo_2_C*T*_*x*_ nanosheets^32^. The observed Mo edge energy is consistent with an average oxidation state of 2D-Mo_2_C*T*_*x*_/SiO_2_ of ca. +5.5 (Fig. 1e, inset and Supplementary Table 1). The reduction of 2D-Mo_2_C*T*_*x*_/SiO_2_ to 2D-Mo_2_C/SiO_2_ in H_2_ leads to the disappearance of the pre-edge feature, as was also observed in the in situ XANES reduction experiment, and shifts the Mo edge positions significantly to lower energies, i.e., from 20015.8 to 20000.8 eV. While the Mo *K*-edge position confirms the carbidic nature of 2D-Mo_2_C/SiO_2_, the white line region of the XANES spectrum of 2D-Mo_2_C/SiO_2_ differs from the spectrum of bulk β-Mo_2_C (Fig. 1e) owing to the different morphologies of these two materials. This observation corroborates the results of TEM analysis and confirms further that sintering to the bulk carbide phase did not occur during the reductive treatment of 2D-Mo_2_C*T*_*x*_/SiO_2_. Consistent with XPS analysis, the XANES spectrum of 2D-Mo_2_CO_*x*_/SiO_2_ reveals a significant shift of the Mo *K*-edge towards higher energies compared to 2D-Mo_2_C/SiO_2_ (i.e., 20015.7 vs 20000.7 eV), suggesting an average Mo oxidation state for 2D-Mo_2_CO_*x*_/SiO_2_ between Mo^5+^ and Mo^6+^ (Fig. 1e inset, Supplementary Table 1). The XANES spectrum of 2D-Mo_2_CO_*x*_/SiO_2_ displays a characteristic pre-edge feature, similarly to 2D-Mo_2_C*T*_*x*_/SiO_2_. We conclude that all available characterization data (XANES, XPS, TEM) are consistent with a predominantly nanosheet morphology and a carbidic and oxycarbidic nature of, respectively, 2D-Mo_2_C/SiO_2_ and 2D-Mo_2_CO_*x*_/SiO_2_.

### Correlating catalytic activity to the surface oxygen coverage of 2D-Mo_2_CO_*x*_

**Fig. 2:**
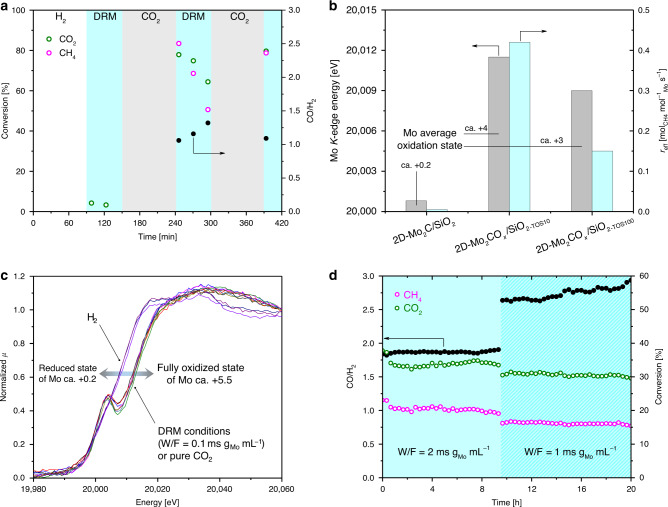
Catalytic performance of 2D-Mo_2_CO_*x*_/SiO_2_ in DRM. **a** Catalytic performance of 2D-Mo_2_C/SiO_2_ (90–150 min) and 2D-Mo_2_CO_*x*_/SiO_2_ (240–300 min and from 390 min after the CO_2_ regeneration step) in DRM at a contact time (W/F) of 3 ms g_Mo_ mL^−1^; **b** correlation of the Mo oxidation state, determined by Mo *K*-edge XANES, and the catalytic activity of the material; **c** Mo *K*-edge XANES spectra of 2D-Mo_2_CO_*x*_/SiO_2_ when exposed to DRM (0.1 ms g_Mo_ mL^−1^ contact time), reductive and oxidative conditions at 730 °C; **d** stable performance of 2D-Mo_2_CO_*x*_/SiO_2_ at contact times of 1 or 2 ms g_Mo_ mL^−1^.

The catalytic activities of 2D-Mo_2_C/SiO_2_ and 2D-Mo_2_CO_*x*_/SiO_2_ for the dry reforming of methane (800 °C, 1 bar, space velocity (SV) 1200 L g_Mo_^−1^ h^−1^, (weight/volume flow rate) W/F = 3 ms g_Mo_ mL^−1^ contact time, 1:1 CO_2_:CH_4_ ratio) are strikingly different (Fig. 2a). While 2D-Mo_2_C/SiO_2_ shows negligible DRM activity, 2D-Mo_2_CO_*x*_/SiO_2_ is highly active with methane conversion rates of 0.42 mol(CH_4_) mol(Mo)^−1^ s^−1^ after 10 min on stream at 80% CH_4_ conversion (i.e., ca. 10% below the thermodynamic equilibrium in those conditions, Fig. 2a)^40^. Note that the 2D nature of our catalysts enables a very high utilization of Mo such that when normalized by weight of Mo, the initial methane consumption rate of 2D-Mo_2_CO_*x*_/SiO_2_ is ~10–200 times higher than the rates that have been reported for other Mo_2_C-based catalysts at similar conditions (Supplementary Table 3). The reference catalysts β-Mo_2_C and *m*-Mo_2_C*T*_*x*_ exhibit a significantly lower DRM activity in these conditions (ca. 0.0003 and 0.002 mol(CH_4_) mol(Mo)^−1^ s^−1^ after ca. 5 min of time in stream, respectively), and deactivated entirely after 30 min of reaction (Supplementary Fig. 17). The CO chemisorption capacity was determined for in situ reduced 2D-Mo_2_C*T*_*x*_/SiO_2_ and the reference β-Mo_2_C catalysts in order to establish if the superior catalytic performance of 2D-Mo_2_C*T*_*x*_/SiO_2_ with respect to β-Mo_2_C (normalized per mass of Mo) is due to the improved utilization of Mo atoms in the 2D-nanosheet catalyst, or due to a higher activity of the Mo sites in 2D-Mo_2_C*T*_*x*_/SiO_2_ owing to their different structure and/or morphology, or a combination of these factors. Our results show that the estimated areal CO capacity in 2D-Mo_2_C*T*_*x*_/SiO_2_ and the reference β-Mo_2_C catalysts are nearly the same (ca. 0.2 and 0.25–0.5 μmol m^–2^, respectively; see Supplementary Table 4 and “Methods” for details). However, the measured methane conversion rate is ca. 3 orders of magnitude higher for 2D-Mo_2_C*T*_*x*_/SiO_2_ relative to the β-Mo_2_C catalyst. These results provide strong evidence that the improved catalytic activity is due to the different nature of Mo sites in 2D-nanosheet catalyst.

When using a CH_4_:CO_2_ feed ratio of 1:1 and a contact time of 3 ms g_cat_ L^−1^, 2D-Mo_2_CO_*x*_/SiO_2_ continuously deactivates, with the methane consumption rate dropping within 100 min on stream by almost a factor of three to 0.15 mol(CH_4_) mol(Mo)^−1^ s^−1^. At this point, the CH_4_ conversion has declined from ca. 80 to 30%. However, the initial activity can be fully regenerated by subjecting the catalyst, after 100 min time on stream (TOS), to a flow of pure CO_2_ at 800 °C for 1 h. While carbide catalysts are generally not known for deactivation by coking^10^, we evaluated the possible deposition of carbon during the DRM catalytic test in a temperature programmed oxidation (TPO) experiment performed in a thermogravimetric analyzer (TGA) with a simultaneous detection of the off gas by mass spectrometer (MS, Supplementary Fig. 19). Noteworthy, no increase in the intensity of the CO_2_ peak was observed for the used catalysts after 10 and 100 min of reaction, 2D-Mo_2_CO_*x*_/SiO_2−TOS10_ and 2D-Mo_2_CO_*x*_/SiO_2−TOS100_ (note: these materials remained light gray before and after catalytic testing). Consistent with this, 2D-Mo_2_CO_*x*_/SiO_2−TOS10_ and 2D-Mo_2_CO_*x*_/SiO_2−TOS100_ did not lose any weight during the TPO experiment. Thus, TPO data provide strong evidence that no detectable carbon deposition occurred on 2D-Mo_2_CO_*x*_/SiO_2_ under the experimental conditions of the DRM tests.

While the activation of metallic DRM catalysts by CO_2_ treatment has been reported before^44^, in particular through the removal of deposited carbon by gasification^45^, the reactivation of carbide-based DRM catalysts by CO_2_ is, to the best of our knowledge, unprecedented and remarkable given that Mo_2_C-based catalysts for DRM are typically oxidized by CO_2_ yielding inactive MoO_2_^11,15^. Note that the reference materials could not be reactivated in CO_2_, which is explained by the formation of MoO_2_ layer covering the active phase (Supplementary Fig. 18). We hypothesized that the deactivation of 2D-Mo_2_CO_*x*_/SiO_2_ with time on stream (Fig. 2a) occurred due to a continuous depletion of the oxygen coverage of the CO_2_-activated molybdenum oxycarbide surface. Therefore, to relate the decrease of the catalytic performance of our Mo oxycarbide catalyst to the oxidation state of Mo, 2D-Mo_2_CO_*x*_/SiO_2_ that has been exposed for 10 or 100 min to DRM conditions was analyzed by XPS and XANES. The deconvolution of the Mo *3d* spectrum of 2D-Mo_2_CO_*x*_/SiO_2−TOS10_ reveals Mo^6+^, Mo^5+^, Mo^4+^, and carbidic Mo states with Mo^4+^ being the main component at 41% (Supplementary Table 2). Note that metallic Mo is absent in the fit of 2D-Mo_2_CO_*x*_/SiO_2−TOS10_ in contrast to 2D-Mo_2_CO_*x*_/SiO_2_ discussed above. This might be due to the facile carburization of Mo^0^ with time on stream or the removal of volatile Mo carbonyl species. The deconvolution of the Mo *3d* spectrum of 2D-Mo_2_CO_*x*_/SiO_2−TOS100_ reveals the same oxidation states as those found in 2D-Mo_2_CO_*x*_/SiO_2−TOS10_ (Mo^6+^, Mo^5+^, Mo^4+^, and carbidic states, Supplementary Fig. 23). However, carbidic Mo is the main component in 2D-Mo_2_CO_*x*_/SiO_2−TOS100_ with a fitted fraction of 45% (Supplementary Table 2). This comparison of 2D-Mo_2_CO_*x*_/SiO_2_ after 10 or 100 min of TOS clearly points at a reduced surface oxygen coverage and therefore a higher fraction of carbidic Mo in the less active catalyst, namely 2D-Mo_2_CO_*x*_/SiO_2−TOS100_. Noteworthy, similarly to the as prepared 2D-Mo_2_C*T*_*x*_/SiO_2_, the XRD pattern of 2D-Mo_2_CO_*x*_/SiO_2-TOS100_ features no Bragg peaks, thereby further confirming that no sintering to crystalline molybdenum oxides or carbides had occurred after this time (Supplementary Fig. 6).

XANES data agree well with the XPS results as the catalyst with the highest activity (2D-Mo_2_CO_*x*_/SiO_2−TOS10_) features a Mo *K*-edge position at 20011.5 eV, which corresponds to an average oxidation state of Mo of +4 (Supplementary Fig. 14 and Fig. 2b). Note that the pre-edge feature in 2D-Mo_2_CO_*x*_/SiO_2_ is well-defined, but poorly resolved in 2D-Mo_2_CO_*x*_/SiO_2−TOS10_, suggesting a change in the geometry of the Mo sites due to the partial reduction during DRM conditions (Supplementary Fig. 14). 2D-Mo_2_CO_*x*_/SiO_2−TOS100_ displays a Mo *K*-edge position at 20009.0 eV, corresponding to an average Mo oxidation state of ca. +3. This means that 2D-Mo_2_CO_*x*_/SiO_2_ does not produce any measurable amount of coke when operated under a contact time of 3 ms g_Mo_ mL^−1^ and deactivates by reduction, lowering the oxygen coverage with TOS compared to the as-CO_2_-treated 2D-Mo_2_CO_*x*_/SiO_2_ material. Subjecting 2D-Mo_2_CO_*x*_/SiO_2−TOS100_ to a CO_2_ atmosphere (800 °C, 1 h) recovers the fully-oxidized 2D-Mo_2_CO_*x*_/SiO_2_ characterized by a Mo oxidation state of ca. +5.5, that is the same as in the as-prepared 2D-Mo_2_CO_*x*_/SiO_2_ material (Supplementary Fig. 14). Likewise, the identical XANES spectra of 2D-Mo_2_CO_*x*_/SiO_2_ prior to DRM testing and after a CO_2_-based regeneration step are also in line with a fully recovered oxygen coverage after regeneration (Fig. 2a and Supplementary Fig. 14). TEM images of the regenerated material reveal that the 2D-Mo_2_CO_*x*_ nanosheets have a similar morphology to the initial 2D-Mo_2_C*T*_*x*_ nanosheets as the hexagonal ordering of the Mo atoms and the characteristic scrolling of the nanosheets at the edges are still observed (Supplementary Fig. 10). Overall, these results highlight the remarkable structural stability of silica-supported 2D-Mo_2_CO_*x*_ nanosheets under catalytic DRM environments as well as in an oxidative CO_2_ atmosphere at 800 °C.

To further interrogate the relationship between the oxygen coverage of 2D-Mo_2_CO_*x*_/SiO_2_ and the catalytic activity in DRM, an in situ XANES experiment was performed (BM31, ESRF)^46^ in a capillary cell reactor wherein 2D-Mo_2_C*T*_*x*_/SiO_2_ was first treated under H_2_ (750 °C, 0.5 h) to prepare 2D-Mo_2_C/SiO_2_ followed by exposing the material to DRM conditions at 730 °C (CH_4_: CO_2_ = 1: 1, SV = 3 × 10^4^ L g_Mo_^−1^ h^−1^, contact time of 0.1 ms g_Mo_ mL^−1^). In those DRM conditions, an immediate oxidation of 2D-Mo_2_C/SiO_2_ to 2D-Mo_2_CO_*x*_/SiO_2_ occurred as indicated by a change of the Mo oxidation state from ca. +0.2 to +5 (Mo edge positions of 20000.7 and 20014.4 eV, respectively), consistent with a high oxygen coverage of the material (Supplementary Fig. 23). Monitoring the off-gas composition using a compact gas chromatograph (GC)^47^ shows no catalytic activity at those high oxygen coverages of 2D-Mo_2_CO_*x*_. Hence, no reduction of 2D-Mo_2_CO_*x*_ with TOS occurs at low contact times of the synchrotron experiment. The oxygen coverage of 2D-Mo_2_CO_*x*_/SiO_2_ is high under those DRM conditions as a change to a CO_2_ flow does not affect the XANES spectra significantly (Fig. 2c and Supplementary Fig. 24). Switching to a N_2_ atmosphere and co-feeding ca. 5% of H_2_ instantly reduces Mo to the carbidic state, yielding XANES spectra similar to that of 2D-Mo_2_C/SiO_2_ (Fig. 2c). However, introducing the DRM feed re-forms immediately 2D-Mo_2_CO_*x*_/SiO_2_ with a Mo oxidation state of ca. +5, inactive for the DRM. The major difference between the laboratory (Fig. 2a) and synchrotron experiments lies in the space velocity that is roughly 25 times higher in the synchrotron experiment. We conclude that while the ex situ XANES data associate the highest DRM activity of 2D-Mo_2_CO_*x*_/SiO_2_ with an average Mo oxidation state of +4 and subsequent deactivation with TOS with the reduction of Mo, in situ Mo *K*-edge DRM experiment revealed that the oxycarbidic surface with a high oxygen coverage and an average oxidation state of +5 is inactive for DRM. The latter observation is consistent with the reduction of the average Mo oxidation state from +5.5 to +4 in 2D-Mo_2_CO_*x*_/SiO_2_ within 10 min TOS in a laboratory DRM catalytic test (W/F = 3 ms g_Mo_ mL^−1^). Note that no characteristic MoO_2_ features were observed with both ex and in situ XANES spectra for the catalyst after DRM catalytic tests, indicating that 2D-Mo_2_CO_*x*_/SiO_2_ does not oxidize to bulk Mo oxides in the presence of both CO_2_ and H_2_O (the latter, owing to the parallel reverse water-gas shift (RWGS, CO_2_ + H_2_ ↔ CO + H_2_O) reaction).

Next, we varied the contact time of the laboratory DRM experiment and found that for a contact time between 1 and 2 ms g_Mo_ mL^−1^, the performance of 2D-Mo_2_CO_*x*_/SiO_2_ in DRM is stable over 20 h TOS (Fig. 2d). Note that the measured ratio of CO:H_2_ of ca. 2 and 3 for, respectively, 2 and 1 ms g_Mo_ mL^−1^, indicates that the RWGS reaction takes place as a side reaction under these conditions. Noteworthy, the contact times associated with a stable catalytic performance are intermediate between the contact times used in the synchrotron experiment and laboratory experiments in which catalyst deactivation occurred (ca. 0.1 and 3 ms g_Mo_ mL^−1^, respectively). This suggests that at short contact times, dissociation of CO_2_ proceeds fast and yields a too high oxygen coverage of the Mo_2_CO_*x*_ surface, while at high contact times CH_4_ and/or DRM products (CO and H_2_) deplete the surface coverage of Mo_2_CO_*x*_ beyond the optimal value, leading to catalyst deactivation. Note that 2D-Mo_2_CO_*x*_/SiO_2_ exhibits a high stability over 20 h on stream at W/F = 1–2 ms g_Mo_ mL^−1^ despite the presence of steam produced by the competing RWGS reaction. However, while at these conditions the selectivity and catalytic activity are stable, both values are lower than the initial (viz. TOS = 10 min) activity and selectivity of 2D-Mo_2_CO_*x*_/SiO_2_ at W/F = 3 ms g_Mo_ mL^−1^ (Fig. 2a, d). Our search for a stable catalytic performance with increased activity and selectivity as compared to conditions of W/F = 1–2 ms g_Mo_ mL^−1^ uncovered that performing the catalytic test at a higher pressure (i.e., 8 bar instead of 1 bar) and keeping W/F = 3 ms g_Mo_ mL^−1^ yields a CO:H_2_ ratio close to 1.5 and only a slightly lower methane conversion rate as compared to that at 1 bar (0.42 vs 0.34 mol(CH_4_) mol(Mo)^−1^ s^−1^) (Supplementary Fig. 22). Thus, oxygen coverage higher than optimal (i.e., corresponding to an Mo oxidation state of +4) not only decreases the methane conversion rates, but also compromizes the selectivity due to the competing RWGS reaction.

Finally, aiming to induce major structural changes in 2D-Mo_2_CO_*x*_/SiO_2_, we subjected the material to three subsequent DRM-catalysis-CO_2_-regeneration cycles with a total TOS of ca. 30 h using a contact time of 2 ms g_Mo_ mL^−1^ for the DRM step (Supplementary Fig. 20). Note, that the resulting material collected after the DRM step (labeled 2D-Mo_2_CO_*x*_/SiO_2−spent_) was only partially deactivated, featuring a methane conversion rate of 0.08 mol(CH_4_) mol(Mo)^−1^ s^−1^. Similarly to 2D-Mo_2_CO_*x*_/SiO_2−TOS10_, deconvolution of the Mo *3d* spectrum of the spent catalyst reveals Mo^6+^, Mo^5+^, Mo^4+^, and carbidic Mo components, with Mo^4+^ being the main component at 62% (Supplementary Fig. 23 and Supplementary Table 2). TEM analysis of 2D-Mo_2_CO_*x*_/SiO_2−spent_ (exposed to air) reveals the presence of 2D nanosheets with a hexagonal ordering (Supplementary Fig. 11) and overlapping signals of Mo, C, and O in the EDX maps, confirming the oxycarbidic nature of the catalyst. Additionally, areas containing a bulk crystalline phase of MoO_2_ with a distorted rutile structure were revealed by FT HR-TEM imaging (Supplementary Fig. 11), in agreement with the high fraction of Mo^4+^ identified in the XPS fittings of this sample and its reduced activity relative to the most active 2D-Mo_2_CO_*x*_/SiO_2−TOS10_ catalyst. In agreement with TEM results, the XRD analysis of 2D-Mo_2_CO_*x*_/SiO_2-spent_ revealed small but discernable peaks corresponding to MoO_2_. That said, supported nanosheets remain the dominant morphology of the spent catalyst, underlining its remarkable stability.

### Theoretical modeling of DRM pathways on 2D-Mo_2_CO_*x*_/SiO_2_

**Fig. 3:**
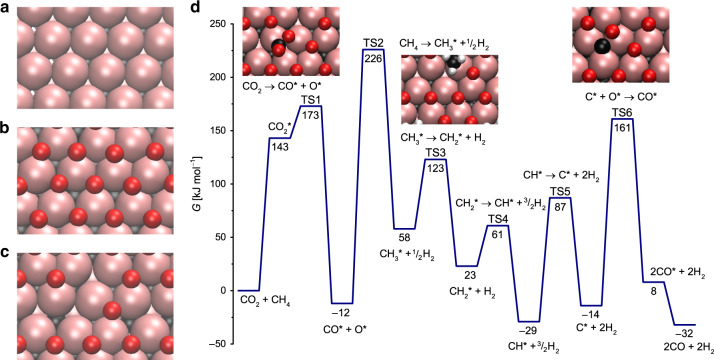
Theoretical modeling of DRM pathways. Top view of the **a** clean 2D-Mo_2_C, **b** 2D-Mo_2_C-1 O ML and **c** 2D-Mo_2_C-0.67 O ML surfaces. **d** Gibbs free energy profile of the dry reforming of methane catalyzed by the 2D-Mo_2_C-0.67 O ML surface. Gibbs energies are given with respect to initial reactants, CH_4_ and CO_2_, at 800 °C and under 1 bar pressure. For brevity, the reaction profile is presented without H–H coupling steps. The full profile is shown in Supplementary Fig. 25. TS stands for transition state.

DFT calculations provided further insight into the role of oxygen coverage for DRM activity and the energetically preferred structure of 2D-Mo_2_C nanosheets after CO_2_ treatment and under DRM conditions. 2D-Mo_2_C was modeled by three atomic layers, with two Mo layers exposed to reactants and a carbon layer in between these Mo layers (Fig. 3). The stability of the orthorhombic Mo_2_C and Mo_2_CO_*x*_ phases has been previously evaluated by DFT calculations^48,49^. We derived 2D-Mo_2_C surface from the crystal structure of Mo_2_Ga_2_C, slicing it along the 001 direction, since it is the most convenient approach to obtain a 2D system with the desired stoichiometry (Supplementary Fig. 29). The computational model used has Mo sites surrounded by three carbon atoms (Mo–C distance of 2.09 Å), with the inner layer of carbon atoms separating the Mo atoms of the top and bottom surfaces by 2.88 Å. The coordination number of the Mo surface atoms is 6; the Mo–Mo distances at the surface are 3.03 Å. Note that EXAFS fitting of 2D-Mo_2_C*T*_*x*_/SiO_2_, as discussed above, revealed that Mo is coordinated with O and with C/O at a distance of ca. 1.74–2.03 Å, which is close to the 2.09 Å Mo–C distance of the theoretical model (Supplementary Table 3). The EXAFS analysis revealed Mo–Mo distances in the range of 2.63–3.42 Å (Table 1); this splitting of distances, as compared with the single Mo–Mo shell of the ideal model, is an indication of lattice strain in the Mo_2_C*T*_*x*_ sheets. We note that our idealized model does not contain lattice strain or curvature of the experimental 2D-Mo_2_CO_*x*_/SiO_2_ nanosheets, and the model does not include the interaction of 2D-Mo_2_CO_*x*_ with dehydroxylated SiO_2_ (i.e., covalent grafting observed by IR spectroscopy). Additionally, the average Mo oxidation state at one monolayer (ML) oxygen coverage of our model is lower than the experimentally determined (by XANES) Mo oxidation state for 2D-Mo_2_CO_*x*_/SiO_2_ (ca. +5.5, Fig. 1e and Supplementary Table 5). Thus, it is likely that 2D-Mo_2_CO_*x*_/SiO_2_ contains more oxygen atoms than 1 O ML. That being said, our idealized model is able to rationalize the experimental trends and it provides insights on the oxygen coverage (and the oxidation state of Mo), which has a significant impact on the DRM catalytic activity. Our evaluation of the thermodynamic stability of the different oxygen coverages of the 2D-Mo_2_C slab at 800 °C under CO_2_ reveals that a molybdenum oxycarbide phase with one oxygen monolayer (1 O ML) adsorbed on each of the Mo layers, i.e., 2D-Mo_2_CO_2_, is preferred thermodynamically (see the Supplementary computational details for further details). 2D-Mo_2_CO_2_ is formed from 2D-Mo_2_C and CO_2_ that react yielding one oxygen atom chemisorbed on the carbide surface with a concomitant release of one gas-phase CO molecule. To reach this full oxygen surface coverage, 18 CO_2_ molecules react per unit cell (9 CO_2_ molecules per each side of the slab) in a highly exergonic reaction releasing Gibbs energy of −928 kJ mol^−1^ per unit cell (ca. −52 kJ mol^−1^ per CO_2_ molecule). The reaction of an additional CO_2_ molecule yields an overall Gibbs free energy of −903 kJ mol^−1^, therefore, this step is unfavorable and the most stable configuration corresponds to 1 O ML coverage (i.e. one oxygen atom per Mo atom on the 2D-Mo_2_C surface), which is in agreement with the observed stability of 2D-Mo_2_CO_*x*_/SiO_2_ in a CO_2_ atmosphere at 800 °C. Under DRM conditions, the reaction of 2D-Mo_2_C 1 O ML with one methane molecule has favorable energetics for the formation of one CO and two H_2_ gas-phase molecules, reducing thereby the surface oxygen coverage. Specifically, decreasing the oxygen coverage of 2D-Mo_2_CO_*x*_ from a full coverage of 1 O ML to 0.89, 0.78, and 0.67 O ML proceeds with reaction energetics of −30, −18 and 0 kJ mol^−1^, that is changing from exergonic to isoergonic energetics. This data confirm that the full coverage of the surface by 1 O ML is thermodynamically unstable under DRM conditions, as observed experimentally.

In the following, we selected 2D-Mo_2_C-0.67 O ML as a starting point to calculate the energy profile of the DRM reaction pathways since the oxygen coverage of this structure is consistent with the optimal oxygen coverage observed experimentally (characterized by an average Mo oxidation state of +4). This partial 0.67 O ML oxygen coverage allows for free reactive Mo sites on the surface. The energetic penalty to form 2D-Mo_2_C-0.67 O ML from 2D-Mo_2_CO_2_ is merely +30 kJ mol^−1^ higher than forming the most stable oxygen coverage under the DRM conditions, i.e., 2D-Mo_2_C-0.88 O ML. The adsorption of CO_2_ on 2D-Mo_2_C-0.67 O ML is endergonic by 143 kJ mol^−1^, leading to a bent CO_2_ on the Mo sites of the oxycarbide surface. Dissociation of this bent CO_2_ to adsorbed CO* and O* products requires 173 kJ mol^−1^ of Gibbs free energy, and is overall exergonic by 12 kJ mol^−1^ (Fig. 3). This Gibbs energy barrier is even lower than the one obtained for a Ni(111) surface at 650 °C (209 kJ mol^−1^)^50^. In terms of required electronic energy, CO_2_ cleavage of the adsorbed, bent CO_2_ intermediate has a very low energy barrier of +31 kJ mol^−1^. This bent CO_2_ adsorbate was evaluated previously on several metal carbides^51^ and was suggested to enhance the reactivity of CO_2_ on interfaces between a metal and a metal carbide^52^ or a metal oxide^53–55^. DFT calculations on the clean, Mo-terminated surface of δ-Mo_2_C(001) showed that CO_2_ activation on molybdenum carbides is also associated with low activation barriers^56^. In turn, the activation of CH_4_ via C–H bond cleavage requires a higher Gibbs activation energy of 242 kJ mol^−1^ and the reaction occurs on Mo sites of the oxycarbide surface. The calculated energy barrier is comparable to the ones computed for Ni(111), Pd(111), and Pt(111) surfaces^50^. Conversely, CH_4_ activation on oxygen sites to form *OCH_3_ and *OH is endergonic by 105 kJ mol^−1^, which is by 45 kJ mol^−1^ less favorable than CH_4_ activation on the Mo sites of the same surface.

Subsequent CH_3_ and CH_2_ activation steps to form CH_2_* and CH* species are significantly less energy demanding than the initial C–H activation of CH_4_, with respective Gibbs energy barriers of 63 and 38 kJ mol^−1^. However, the cleavage of the C–H bond in CH* to form adsorbed C and H has a high Gibbs energy barrier of 117 kJ mol^−1^. Note that each successive C–H activation of methane generates an adsorbed hydrogen that can be released as an H_2_ in a strongly entropically favored process, releasing 59 kcal mol^−1^ at 800 °C per each desorbed H_2_ molecule (Fig. 3).

Besides the activation of CO_2_ and CH_4_, another key step in the dry reforming of methane is the oxidation of either CH* or C* species on the oxycarbide surface by adsorbed oxygen (O* or structural oxygen)^57^ ultimately producing adsorbed CO. In the case of 2D-Mo_2_CO_*x*_, and in contrast to metallic surfaces^50^, the preferred mechanism is the direct oxidation of adsorbed C* by adsorbed O* to form CO*. This pathway has a Gibbs energy barrier of 175 kJ mol^−1^, and it is endergonic by only 22 kJ mol^−1^.The alternative pathway involving the coupling of CH* and O* to form adsorbed CHO*, followed by its decomposition to co-adsorbed CO* and H*, is less favorable (Supplementary Fig. 26). Finally, desorption of CO is exergonic by 20 kJ mol^−1^ per CO molecule. Overall, the calculated energy barriers of the proposed mechanism are feasible under the DRM conditions studied here (800 °C, CH_4_: CO_2_ = 1:1) and are thermodynamically exergonic by 32 kJ mol^−1^. The most energy demanding step is the initial methane activation step, which is associated with a significantly higher barrier than the direct CO_2_ activation or the C–O coupling step forming CO. In addition, the relative rates for the formation and consumption of intermediate oxygen and carbon surface species, and, therefore their ratio on the 2D-oxycarbide surface, has to be similar in order to obtain a good catalytic performance. Our theoretical calculations suggest that the ratio of C* to O* is another possible factor (besides the oxidation state of Mo atoms on the surface) that can contribute to the significantly higher catalytic activity of the 2D-Mo_2_C-0.67 O ML system in comparison to the clean 2D-Mo_2_C surface.

**Fig. 4:**
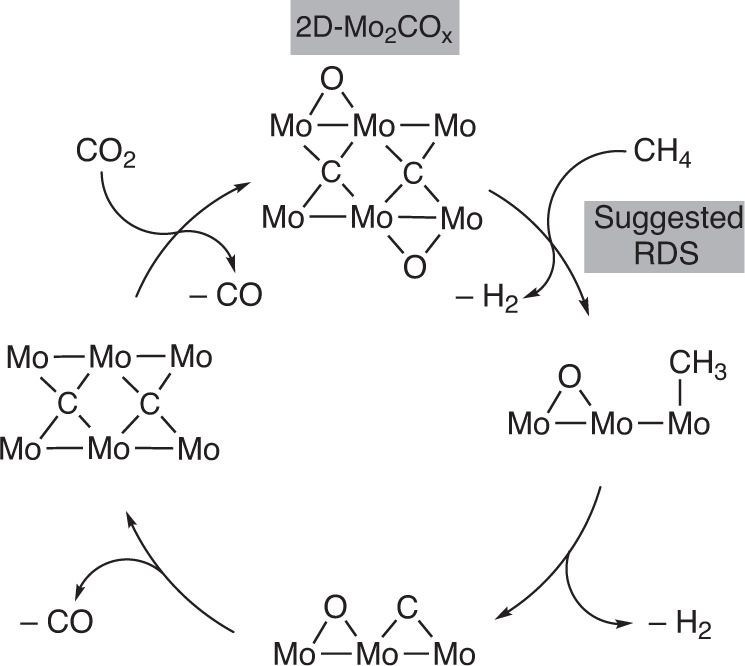
A simplified proposed mechanism for the dry reforming of methane on 2D-Mo_2_CO_*x*_. The most active catalyst has a partial oxygen surface coverage, corresponding to Mo(+4) oxidation state. The C–H bond activation of methane is proposed as RDS, i.e., rate determining step, based on DFT calculations.

We also evaluated the energy profile of the DRM reaction on a clean 2D-Mo_2_C surface and found that the activation of CO_2_ occurs on this surface easier than on 2D-Mo_2_C-0.67 O ML. The produced CO* and O* surface species are significantly more stabilized on 2D-Mo_2_C compared to 2D-Mo_2_C-0.67 O ML, featuring a lower Gibbs energy barrier for CO_2_ activation of 109 kJ mol^−1^ (compare to 173 kJ mol^−1^ for the 2D-Mo_2_C-0.67 O ML surface). The resulting adsorbed CO* and O* species are located at −99 kJ mol^−1^ for the clean 2D-Mo_2_C surface, which is significantly lower than for the 2D-Mo_2_C-0.67 O ML surface (−12 kJ mol^−1^, Supplementary Fig. 27). That said, the free energy barrier for CH_4_ activation is very similar for both surfaces (238 vs. 243 kJ mol^−1^). Another difference with implications for the catalytic activity is the substantial stabilization of CH* and C* species on the 2D-Mo_2_C surface with respect to the initial reactants. The high adsorption energy of C* and O* species lead to a significantly higher energy barrier for the C–O coupling step (+219 kJ mol^−1^) on 2D-Mo_2_C, in contrast to the respective barrier on the 2D-Mo_2_C-0.67 O ML surface (+175 kJ mol^−1^). This overstabilization of CH* and C* and O* intermediates on a clean 2D-Mo_2_C surface explains its lower activity for DRM. In contrast, the 2D-Mo_2_C-0.67 O ML surface does not bind intermediates too strongly. Figure 4 summarizes our computational studies, which agree well with the experimental results discussed above.

To conclude, we have shown that supporting and dispersing delaminated 2D-Mo_2_C*T*_*x*_ nanosheets on silica prevents to a large extent their thermal sintering to bulk Mo_2_C and MoO_2_ phases, paving the way for the application of 2D Mo-carbides and oxycarbides for high temperature heterogeneous catalysis. XANES and XPS analysis indicate that oxycarbidic 2D-Mo_2_CO_*x*_ with an average Mo oxidation state of +4 is the active phase for DRM and oxidizing or reducing this state reduces the methane consumption rate. DFT calculations suggest that the rate-limiting step in the DRM on 2D-Mo_2_CO_*x*_ is CH_4_ cleavage and that the oxygen coverage resulting from the initial CO_2_ activation steps reduces the binding energy of C* and O* species and lowers, in turn, the energy barriers of the C–O coupling step. Importantly, when deactivation by reduction of the oxygen coverage has occurred, the catalyst can be reactivated to its initial activity by re-oxidation in a pure stream of CO_2_. Noteworthy, the nanosheet morphology of 2D-Mo_2_CO_*x*_ is maintained during catalysis and regeneration. In addition, we identified hydrodynamic conditions that ensured a stable performance over 20 h time on stream, owing to an in situ regeneration of the optimal oxygen coverage in those conditions. Overall, the results obtained indicate that supported 2D-Mo_2_CO_*x*_ is a very active and robust catalyst for high temperature catalytic applications, exhibiting a remarkable stability in oxidative environments.

## Methods

### Materials

Multi-layered Mo_2_C*T*_*x*_ material (*m***-**Mo_2_C*T*_*x*_) was synthesized following a reported method^32^. 2D-Mo_2_C*T*_*x*_ nanosheets were delaminated from *m-*Mo_2_C*T*_*x*_ (50 mg) in ethanol (10 mL) using a pulse sonication (1.5 h, 85 W, 20 kHz) (Fisher Scientific, FB120). Resulting suspension was centrifuged (7000 rpm, 7 min) and the supernatant solution containing colloidal delaminated Mo_2_C*T*_*x*_ nanosheets (*d-*Mo_2_C*T*_*x*_) was further used for the incipient wetness impregnation (IWI) onto SiO_2_ support (150–300 μm particle size fraction of Aerosil 300 that had been calcined at 950 °C, 194 m^2^ g^−1^ surface area by nitrogen physisorption). The IWI procedure using ethanol colloidal solution of *d-*Mo_2_C*T*_*x*_ was repeated multiple times and after drying (100 °C, 12 h) yielded 2D-Mo_2_C*T*_*x*_/SiO_2_ material with Mo loading of 0.48 wt.% (batch 1) and 0.35 wt.% (batch 2) according to elemental analysis (ICP-AES). 2D-Mo_2_C*T*_*x*_/SiO_2_ was subsequently reduced in hydrogen (20 vol % H_2_/N_2_, 800 °C, 1.5 h, 50 mL min^−1^, 10 °C min^−1^) and oxidized in CO_2_ (100% CO_2_ 10 mL min^−1^, 800 °C, 1.5 h) to give 2D-Mo_2_C/SiO_2_ and 2D-Mo_2_CO_*x*_/SiO_2_ materials, respectively. To avoid surface modification of the activated materials, 2D-Mo_2_C/SiO_2_, 2D-Mo_2_CO_*x*_/SiO_2_, and all activated catalysts were transferred into a nitrogen-filled glovebox (H_2_O and O_2_ levels <0.5 ppm) and handled without exposure to air.

### Characterization

Ex situ XRD data were examined within the 2*θ* range of 5–90° (step size and scan time per step were 0.0167° and 3 s, respectively). XRD data were collected on a PANalytical Empyrean X-ray diffractometer equipped with a Bragg–Brentano HD mirror and operated at 45 kV and 40 mA (Cu *K*α radiation, *λ* = 1.5418 nm). TGA was performed on Mettler Toledo TGA/DSC 3 instrument. The concentration of 2D-Mo_2_C*T*_*x*_ flakes in the colloidal solutions was determined by drying a 750 μL aliquot in a sapphire crucible (900 μL) at 80 °C for 1 h (5 °C min^−1^). TPO was performed on Mettler Toledo TGA/DSC 1 instrument. Mo material (20 mg) was placed in an alumina crucible (70 μL) and heated to 800 °C under air flow (10 °C min^−1^, 125 mL min^−1^). The H_2_O and CO_2_ content in the outlet gas was followed by a mass spectrometer MKS Cirrus 3. Inductively coupled plasma-atomic emission spectroscopy (ICP-AES) analyses were performed in Remagen, Germany, by the Mikroanalytisches Labor Pascher. XPS data were acquired using a Sigma II instrument of Thermo Fisher Scientific, equipped with an UHV chamber (non-monochromatic 200 W Al *K*α source, a hemispherical analyzer, and a seven channel electron multiplier, with the analyzer-to-source and the emission angles of 50° of 0°, respectively). The survey and narrow scans used a pass energy of 50 eV and 25 eV, respectively, and C *1s* peak of adventitious carbon was set at 284.8 eV to compensate for charge induced shifts. We used a home-build air-tight cell to transfer reduced or used specimens between the glovebox and the XPS instrument^58^. CasaXPS Version 2.3.19PR1.0 software was used to analyze XPS data. The background was subtracted according to Shirley^59^, and the application of the atomic sensitivity factors (ASF) of Scofield allowed to estimate the atomic composition^60^. HAADF STEM images were recorded using FEI Talos F200X with the accelerating voltage of 200 kV. All samples were prepared by shaking the holey carbon TEM grid in the vial with the dry specimen powder. 2D-Mo_2_C/SiO_2_ and 2D-Mo_2_CO_*x*_/SiO_2_ were deposited on TEM grids in the glove box and transferred to the microscope without ambient exposure by using the TEM vacuum transfer holder (Fischione, 2560). FTIR Spectroscopy was conducted using a 200 mL glass reactor with IR-transparent CaF_2_ windows. A thin pellet of the sample was pressed in a glovebox and loaded to the reactor using a glass sample holder. A spectrum was recorded in an argon atmosphere in a transmission mode on a Nicolet 6700 FTIR spectrophotometer using 64 scans at a resolution of 2 cm^−1^. CO chemisorption was performed using an AutoChem system (Micromeritics) with a thermal conductivity detector (TCD). Approximately 100 mg of the material was loaded in a U-shape reactor and reduced at 800 °C under 5% H_2_/Ar for 1.5 h (10 °C min^−1^, 50 mL min^−1^). The sample was then cooled down under a He atmosphere to 450 °C and degassed for 2 h. The first CO adsorption isotherm was carried out at 50 °C. The reactor was then purged with He for 30 min to remove weakly adsorbed species prior to performing the second CO isotherm, also at 50 °C. The amount of strongly adsorbed CO was determined as a difference between the first and the second adsorption isotherms, which provided the CO adsorption capacity. The surface area of the 2D-Mo_2_C nanosheets in 2D-Mo_2_C/SiO_2_ was estimated based on the crystal structure of Mo_2_C*T*_*x*_^32^. Considering the hexagonal ordering of the Mo atoms with a Mo–Mo distance of 2.8629 Å gave an area estimate of 7.098 Å^2^ per Mo atom. Based on the Mo content in 2D-Mo_2_C*T*_*x*_/SiO_2_, determined by ICP, and the mass of the 2D-Mo_2_C*T*_*x*_/SiO_2_ specimen used for CO chemisorption, the amount of strongly chemisorbed CO was normalized per the surface area of the two-dimensional molybdenum nanosheets (Supplementary Table 4).

Experiments using X-ray absorption spectroscopy (XAS) were performed at the Swiss-Norwegian Beamlines (SNBL, BM31)^46^ at the European Synchrotron Radiation Facility (ESRF, Grenoble, France). A double-crystal Si (111) monochromator with continuous scanning in transmission mode (unfocused beam, 1–2 ∙ 10^9^ ph s^−1^ mm^−1^) was used to collect XAS spectra at the Mo *K*-edge. Edge position of the Mo foil set at 20000 eV served for calibration of the XAS data. A quartz capillary reactor (outer diameter 1.5 mm, wall thickness 0.1 mm) was used to perform the in situ DRM experiment (Supplementary Fig. 1)^46^. 2D-Mo_2_C*T*_*x*_/SiO_2_ (ca. 2 mg), placed between two quartz wool plugs in a quart capillary reactor, was reduced in 20 vol% H_2_ in N_2_ (10 mL min^−1^ total flow rate, 50–750 °C the temperature range, ca. 9 °C min^−1^, 20 min holding time). The oxidation was performed in pure CO_2_ (10 mL min^−1^, 730 °C, 2 h). DRM catalytic experiments were performed at 730 °C using a mixture of CH_4_ and CO_2_ of varied ratio diluted in N_2_ (5 mL min^−1^ total flow rate, SV ca. 30000 L g_Mo_^−1^ h^−1^). The composition of the outlet gas was followed by a portable GC (Global Analyser Solutions) with TCD and FID detectors. Ex situ XAS data were collected from pellets with an optimized amount of specimen mixed with cellulose. All reduced materials or used catalysts derived from 2D-Mo_2_C/SiO_2_ were handled in a glovebox and analyzed in an air-tight sealed bags. XAS data were processed using the Athena software, and EXAFS data were fitted using the Artemis software (Demeter 0.9.25 software package)^61^. The edge position for Mo and Mo_2_C was defined as the position of the first maximum of the first derivative curve. The second maximum of the first derivative curve was chosen as the edge position for oxide and oxycarbide materials.

### Catalytic testing

The catalytic testing of freshly-prepared materials in conditions of the dry reforming of methane was carried out in a fixed-bed quartz reactor at atmospheric pressure. In a typical experiment, 100 mg of freshly prepared 2D-Mo_2_C*T*_*x*_/SiO_2_ material was used. Prior to the activity tests, 2D-Mo_2_C*T*_*x*_/SiO_2_ was in situ reduced in 20 vol % H_2_/N_2_ (800 °C, 1.5 h, 50 mL min^−1^, 10 °C min^−1^) and then oxidized in pure CO_2_ (800 °C, 1.5 h, isothermal). The activity test was then performed at 800 °C with a variable total flow rate of the feed (10–50 ml min^−1^, SV = 1200–6000 L g_Mo_^–1^ h^–1^, 45% CH_4_, 45% CO_2_, and 10% N_2_). The composition of the off-gas was analyzed via a GC (PerkinElmer Clarus 580) equipped with a thermal conductivity TCD detector. The carbon balance during the catalytic tests was generally close to 100 %, maximal deviations noted were ±10%, observed when switching gases.

We note that catalytic results reported were obtained with freshly-prepared 2D-Mo_2_C*T*_*x*_/SiO_2_. When as prepared 2D-Mo_2_C*T*_*x*_/SiO_2_ was left for a prolonged time (1 month–1.5 years) in air, a complete or partial fragmentation of 2D-Mo_2_C*T*_*x*_ nanosheets into nanoparticles had occurred according to TEM characterization (Supplementary Fig. 7). The fully fragmented material is inactive in DRM, which emphasizes the importance of 2D nanosheets morphology for the high catalytic activity.

### Computational details

The Vienna Ab Initio Simulation Package (VASP) was utilized to conduct the periodic DFT calculations^62–64^. The projector-augmented-wave (PAW) method was used, where pseudopotentials describe interactions between valence electrons and ion cores while the electronic wave functions are expanded as a discrete plane wave (PW) basis set^65^. All calculations utilized a PW energy cutoff of 500 eV. The generalized gradient approximation (GGA), including dispersion corrections by the BEEF-vdW density functional, were used to treat electron exchange and correlation^66^.

The sampling of the Brillouin zone was performed using Monkhorst–Pack grids^67^ with K-point values equal to 3 × 3 × 1 for the Mo_2_C surface. Electronic occupancies were determined according to a Gaussian Smearing (with a smearing value of 0.05 eV). Self-consistent field (SCF) calculations of the electronic structure were considered converged once the electronic energy change between two consecutive steps was below 10^−5^ eV. All geometries were optimized fully, i.e., until the forces acting on each atom were converged below 10^−4^ eV Å^−1^. Dipole corrections were applied in the *z*-direction and placed in the center in the unit cell. The energy of isolated molecules was determined by a Γ-point calculation such that each species was placed in a box with dimensions 15 × 15.5 × 16 Å. The climbing image nudge elastic band (CI-NEB) method allowed locating transition states (TS) using eight intermediate images^68^. A frequency analysis confirmed further that identified TS structures corresponded to saddle points. Normal vibration modes of adsorbed species were calculated by diagonalization of the Hessian matrix, obtained using a central finite difference approximation with displacements equal to 0.015 Å in the direction of each Cartesian coordinate. All Mo and C atoms from the carbide were kept fixed during the frequency calculations of the adsorbed species. All the reported energy values in the main text correspond to Gibbs Energies at 800 °C. The theoretical model of the (001) facet of 2D-Mo_2_C was constructed as presented in Supplementary Fig. 29. The experimental bulk structure of Mo_2_Ga_2_C was taken as a starting point. Then, the Ga atoms (in green) were removed from this structure to generate the bulk Mo_2_C structure. Next, an ideal 2D-facet model for Mo_2_C was obtained by selecting the inner fragment of the cell in the 001 direction (Supplementary Fig. 29). This was followed by the full geometry optimization of the resulting structure. The Mo_2_C (001) surface slab were taken with dimensions equal to 3 × 3, using two Mo layers on the top and the bottom of the slab and one middle carbon layer. 15 Å of vacuum were added in the direction perpendicular to the surface.

## Supplementary information

Supplementary Information

## Data Availability

The data supporting the findings of this study are available from the corresponding authors upon reasonable request.
