# Supplementary material for: Exploiting two-dimensional morphology of molybdenum oxycarbide to enable efficient catalytic dry reforming of methane
Source: Nat Commun. 2020 Oct 2;11:4920. doi: 10.1038/s41467-020-18721-0 (PMC7532431; doi:10.1038/s41467-020-18721-0)
Supplement: Supplementary file 1 — Supplementary Information [file 41467_2020_18721_MOESM1_ESM.pdf]

Supplementary Information

Exploiting Two-Dimensional Morphology of Molybdenum Oxycarbide to Enable Efficient Catalytic Dry Reforming of Methane

Alexey Kurlov,[†] Evgeniya B. Deeva,[†] Paula M. Abdala,[†] Dmitry Lebedev,[‡,#] Athanasia Tsoukalou,[†] Aleix Comas-Vives,[*,⊥] Alexey Fedorov,[*,†] Christoph R. Müller[*,†]

† *ETH Zürich, Department of Mechanical and Process Engineering, Leonhardstrasse 21, CH 8092 Zürich, Switzerland*

‡ *ETH Zürich, Department of Chemistry and Applied Biosciences, Vladimir-Prelog-Weg 1-5, CH-8093, Zürich, Switzerland.*

⊥ *Autonomous University of Barcelona, Department of Chemistry, 08193 Cerdanyola del Valles, Catalonia, Spain*

Present address: Department of Materials Science and Engineering, Northwestern University, Evanston, Illinois 60208, United States.

E-mail:

* fedoroal@ethz.ch
* muelchri@ethz.ch
* Aleix.Comas@uab.cat

List of Figures

Supplementary Figure 1. Experiment setup for the in situ experiments performed at SNBL. 4

Supplementary Figure 2. XRD pattern and SEM images of synthesized m-Mo_2CT_x. 4

Supplementary Figure 3. TEM images of exfoliated 2D-Mo_2CT_x. 5

Supplementary Figure 4. XRD pattern of m-Mo_2CT_x and delaminated 2D-Mo_2CT_x. 6

Supplementary Figure 5. TEM images of 2D-Mo_2CT_x/SiO_2 7

Supplementary Figure 6. XRD pattern of 2D-Mo_2CT_x/SiO_2, 2D-$Mo_2CO_x/SiO_{2\text{-TOS100}}$ and 2D-$Mo_2CO_x/SiO_{2\text{-spent}}$. 8

Supplementary Figure 7. TEM images of 2D-Mo_2CT_x/SiO_2 aged in air for ca. 1 month. 9

Supplementary Figure 8. TEM images of 2D-Mo_2C/SiO_2. 9

Supplementary Figure 9. TEM images of 2D-Mo_2CO_x/SiO_2. 10

Supplementary Figure 10. TEM images of 2D-$Mo_2CO_x/SiO_{2\text{-regen}}$ 11

Supplementary Figure 11. TEM images of 2D-$Mo_2CO_x/SiO_{2\text{-spent}}$. 12

Supplementary Figure 12. EXAFS fitting of 2D-Mo_2CT_x/SiO_2. 13

Supplementary Figure 13. XANES spectra of 2D-Mo_2CT_x/SiO_2 and reference materials 13

Supplementary Figure 14. XANES spectra of 2D-Mo_2CT_x/SiO_2 after different treatments. 14

Supplementary Figure 15. IR spectrum of 2D-Mo_2C/SiO_2 with a zoomed in area of a [≡Si–H] region. 15

Supplementary Figure 16. Top: XRD pattern of m-Mo_2CT_x before (black) and after (red) oxidation in CO_2 at 800 °C. Inset: In situ XRD pattern of bulk Mo_2CT_x oxidation in CO_2 (20 vol. % CO_2 in N_2, XRD pattern every 50 °C). Bottom: SEM images of Mo_2CT_x annealed at 800 °C in pure CO_2. 16

Supplementary Figure 17. Catalytic performance of β-Mo_2C (left) and m-Mo_2CT_x (right) in DRM. 17

Supplementary Figure 18. XRD patterns of β-Mo_2C and m-Mo_2CT_x after the DRM catalytic test 17

Supplementary Figure 19. A temperature programed oxidation (TPO) of 2D-$Mo_2CO_x/SiO_{2\text{-TOS10}}$ (top) and 2D-$Mo_2CO_x/SiO_{2\text{-TOS100}}$ (bottom) in a TGA followed by MS. 18

Supplementary Figure 20. DRM stability test of 2D-Mo_2CO_x/SiO_2 with several regeneration/deactivation cycles. 19

Supplementary Figure 21. DRM stability test of 2D-Mo_2CO_x/SiO_2 (CH_4 and CO_2 conversion, %) with the corresponding carbon balance determined by GC. 20

Supplementary Figure 22. Catalytic performance of 2D-Mo_2CT_x/SiO_2 in DRM at elevated pressure. 20

Supplementary Figure 23. Deconvolution of ex situ XPS data of the Mo *3d* core levels of 2D-$Mo_2CO_x/SiO_{2\text{–TOS10}}$, 2D-$Mo_2CO_x/SiO_{2\text{–TOS100}}$ and 2D-$Mo_2CO_x/SiO_{2\text{–spent}}$ 21

Supplementary Figure 24. XANES spectra of 2D-Mo_2CT_x derived materials under different conditions representing a complete reversibility between 2D-Mo_2C/SiO_2 and 2D-Mo_2CO_x/SiO_2. 23

Supplementary Figure 25. Pathway to form C* and $2H_2$ from CH_4 involving the H–H coupling steps. Gibbs energies are given with respect to CH_4 and CO_2 (in kJ mol^{-1}) 26

Supplementary Figure 26. Alternative oxidative pathway to form CO via the CHO* and HCO* intermediates. Gibbs energies are given with respect to CH_4 and CO_2 (in kJ mol^{-1}). 27

Supplementary Figure 27. Gibbs energy profile comparing the reactivities of 2D-Mo_2C-0.67 O ML (blue) and 2D-Mo_2C (red) surfaces 27

Supplementary Figure 28. Mo Bader charge dependence on the oxidation state (based on Mo, MoO_2 and MoO_3). 28

Supplementary Figure 29. Illustration of the construction of the theoretical model of the (001) facet of 2D-Mo_2C. 29

List of Tables

Supplementary Table 1. Mo *K*-edge position and calculated Mo oxidation state for 2D-Mo_2CT_x/SiO_2 treated under different conditions and reference materials. 14

Supplementary Table 2. XPS fitting parameters for Mo_2CT_x, 2D-Mo_2CT_x/SiO_2, 2D-Mo_2C/SiO_2, 2D-Mo_2CO_x/SiO_2, 2D-Mo_2CO_x/SiO_2-DRM, β-Mo_2C and MoO_3. 22

Supplementary Table 3. Comparison of the DRM activity of 2D-Mo_2CO_x/SiO_2 with literature data for Mo-based catalysts. 24

Supplementary Table 4. Total and areal CO capacity as well as the specific (Mo_2C) surface area of 2D-Mo_2C/SiO_2 and β-Mo_2C. 24

Supplementary Table 5. Oxidation states of the Mo atoms in the 2D-Mo_2C models with 1 O ML and 0.67 O ML oxygen coverage. 28

Supplementary Table 6. Comparison of the reactivity of 2D-Mo_2CO_x-0.67 O ML with respect to 2D-Mo_2C surface (in kJ mol^{-1}). Energies are given with respect to the initial reactants, CH_4 and CO_2. A star next to the species indicates the related species is adsorbed either on the 2D-Mo_2C or 2D-Mo_2CO_x-surface (*) 29

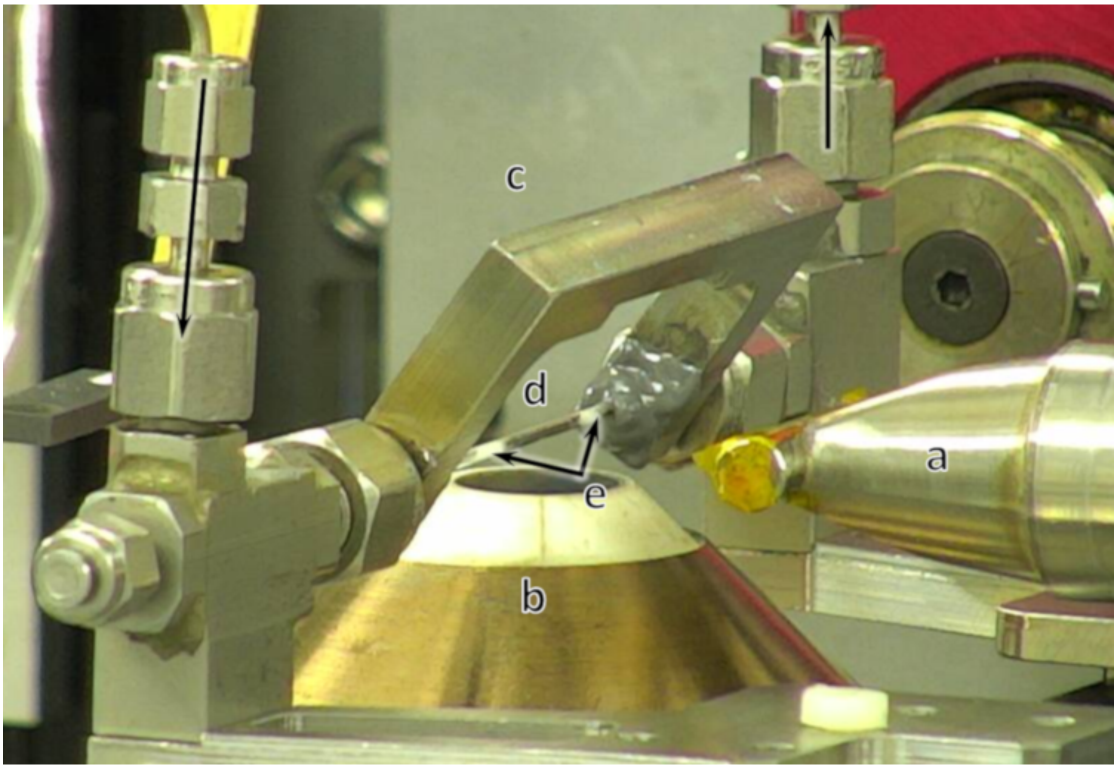

Supplementary Figure 1. Experiment setup for the in situ experiments performed at SNBL.
a) incident beam, b) heating air gun, c) capillary cell, d) sample, e) quartz wool plugs. Arrows indicate the direction of the gas flow.

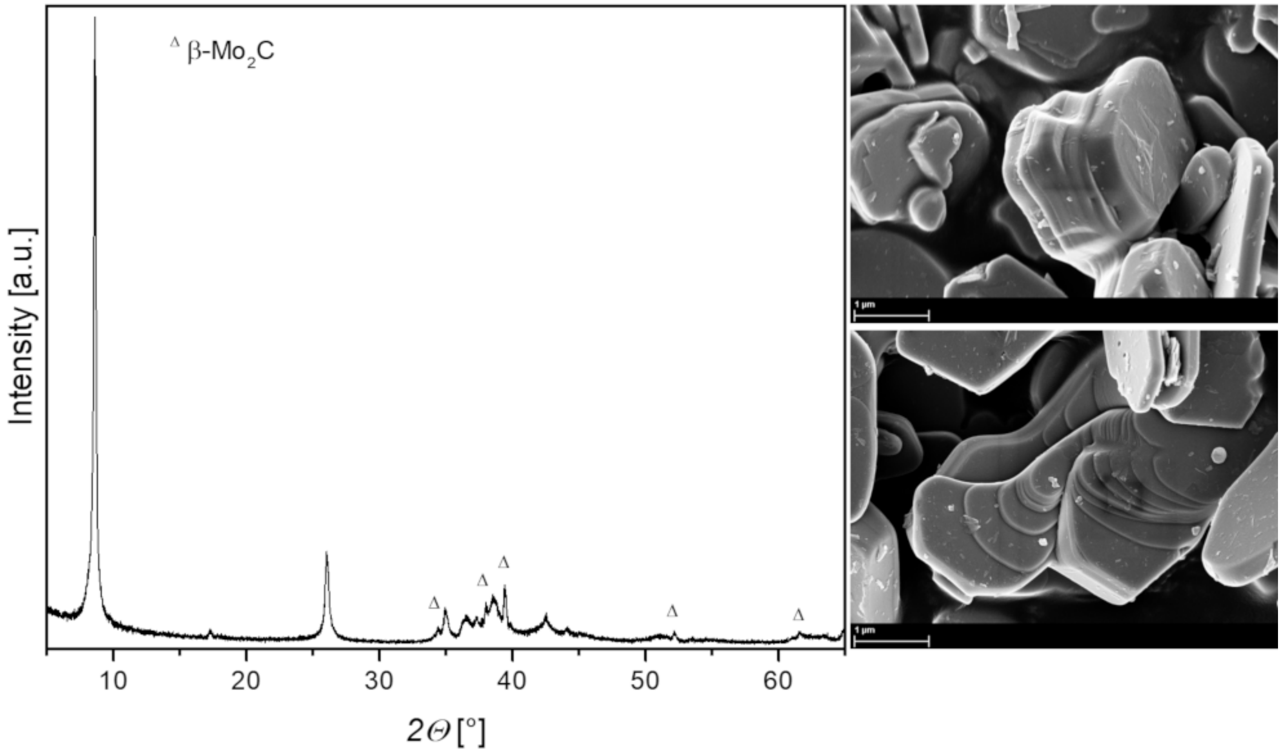

Supplementary Figure 2. XRD pattern and SEM images of synthesized m-Mo_2CT_x.

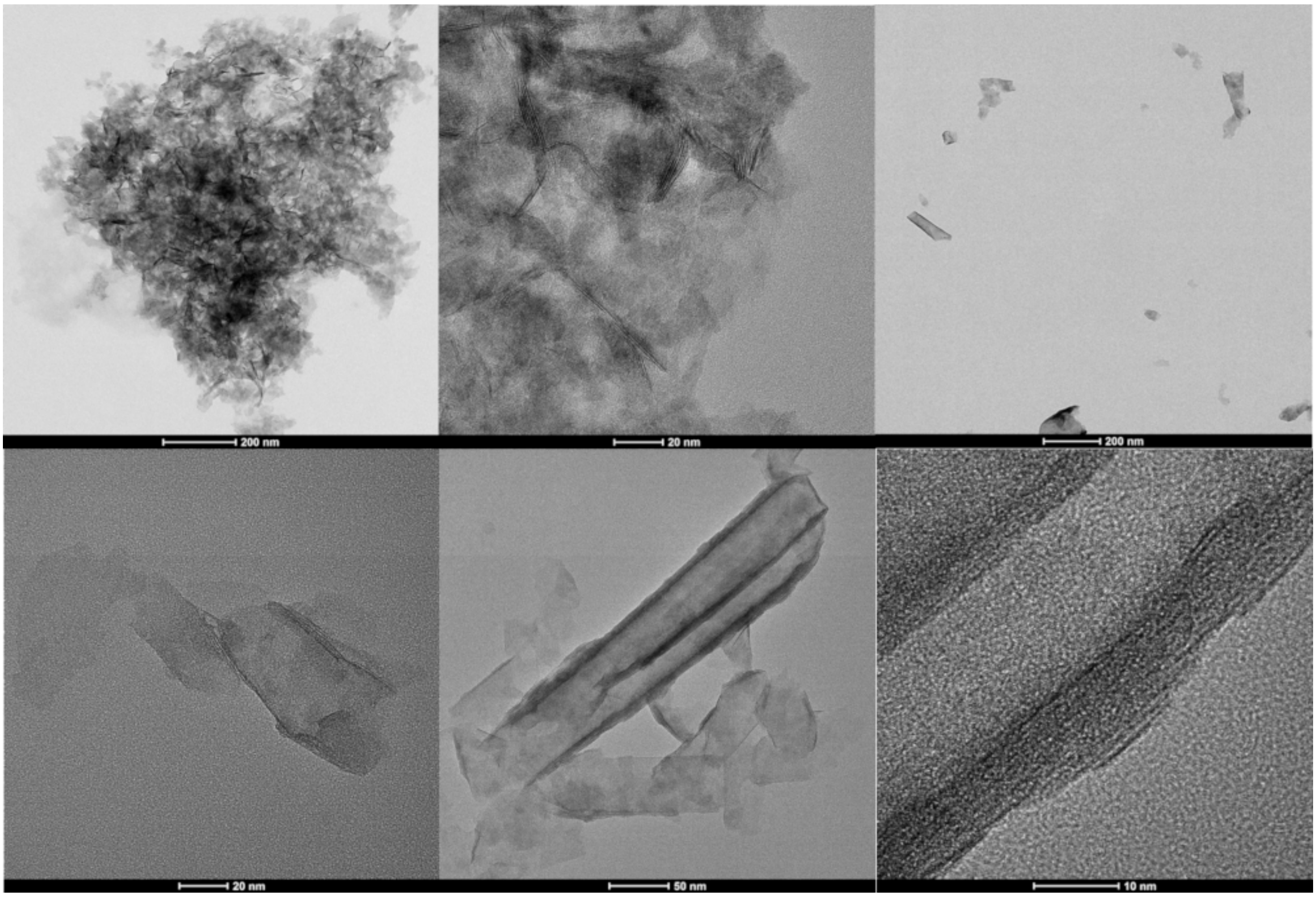

Supplementary Figure 3. TEM images of exfoliated 2D-Mo_2CT_x.

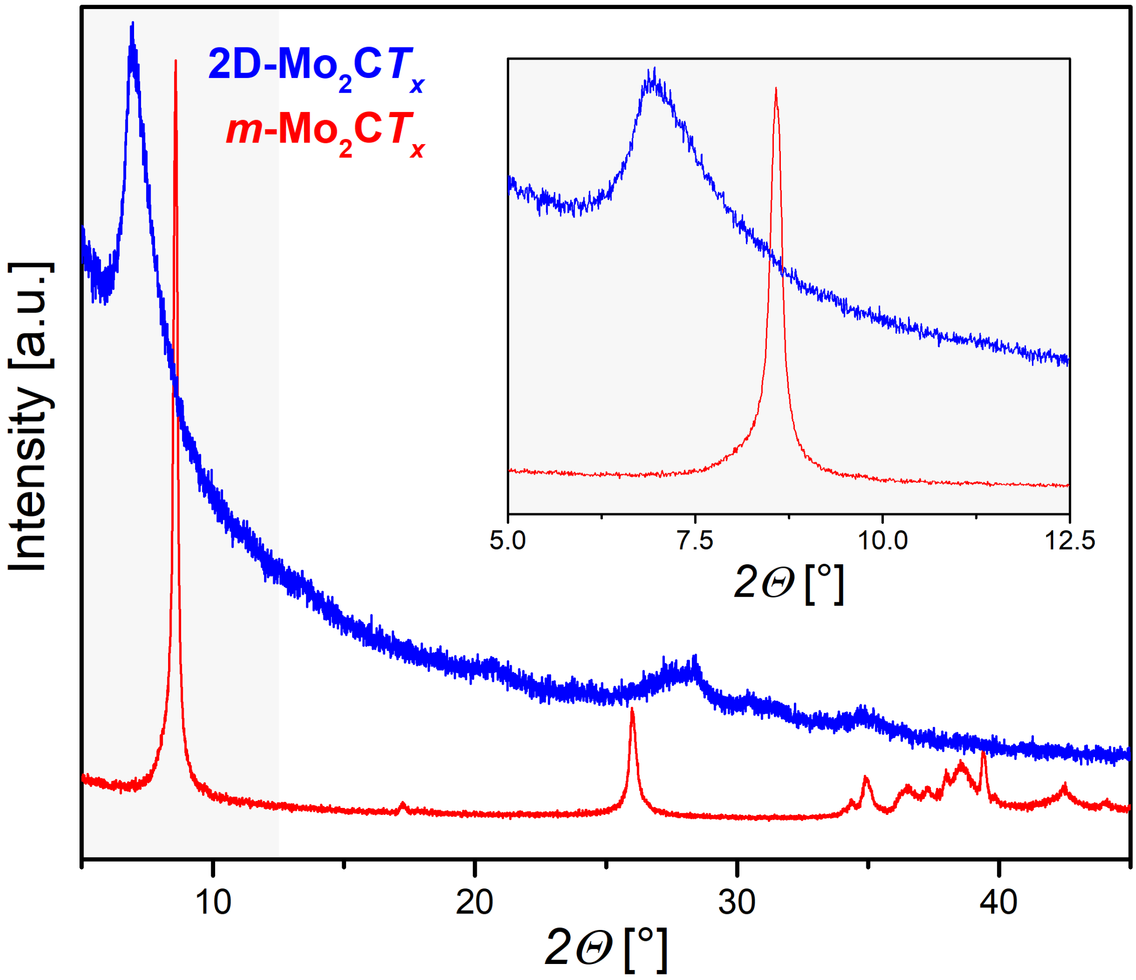

Supplementary Figure 4. XRD pattern of *m*-Mo_2CT_x and delaminated 2D-Mo_2CT_x.

X-Ray powder diffraction pattern of the dried delaminated Mo_2CT_x is similar to that of *m*-Mo_2CT_x material, although with significantly broader peaks shifted towards smaller 2θ values relative to peaks of non-delaminated Mo_2CT_x (Supplementary Figure 4). The shift towards smaller 2θ values can be related to the increase of the interlayer distance from ca. 7 Å in *m*-Mo_2CT_x material to ca. 10 Å in *d*-Mo_2CT_x, caused by the intercalation of ethanol molecules in between the individual 2D-Mo_2CT_x layers. The broadening of the peak at $2\theta = 7$ ° is explained by the loss of coherence (more disordered structure of the nanosheets) during delamination of individual 2D-Mo_2CT_x flakes and their non-regular re-stacking upon drying of the solution with *d*-Mo_2CT_x.

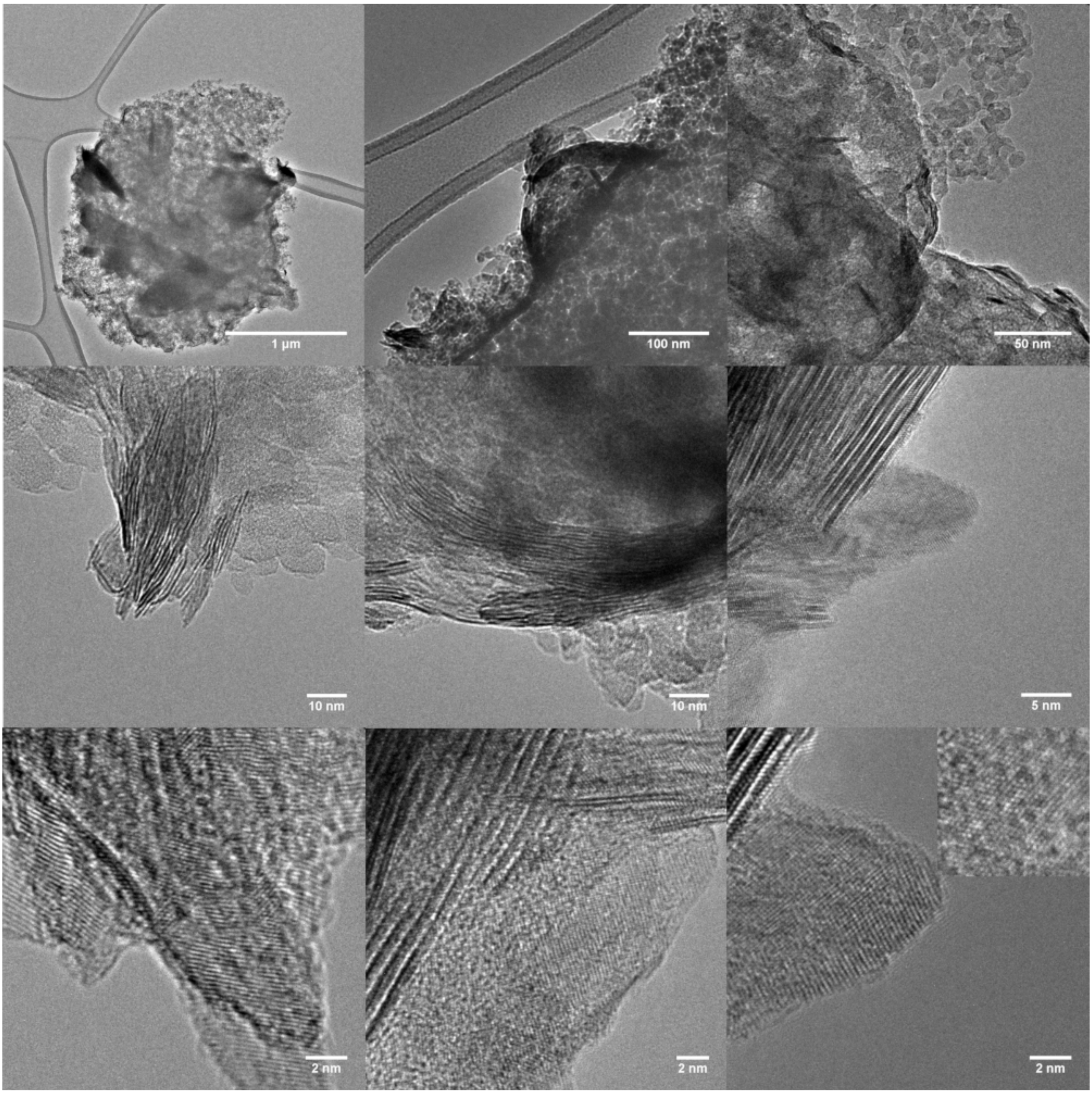

Supplementary Figure 5. TEM images of 2D-Mo_2CT_x/SiO_2.

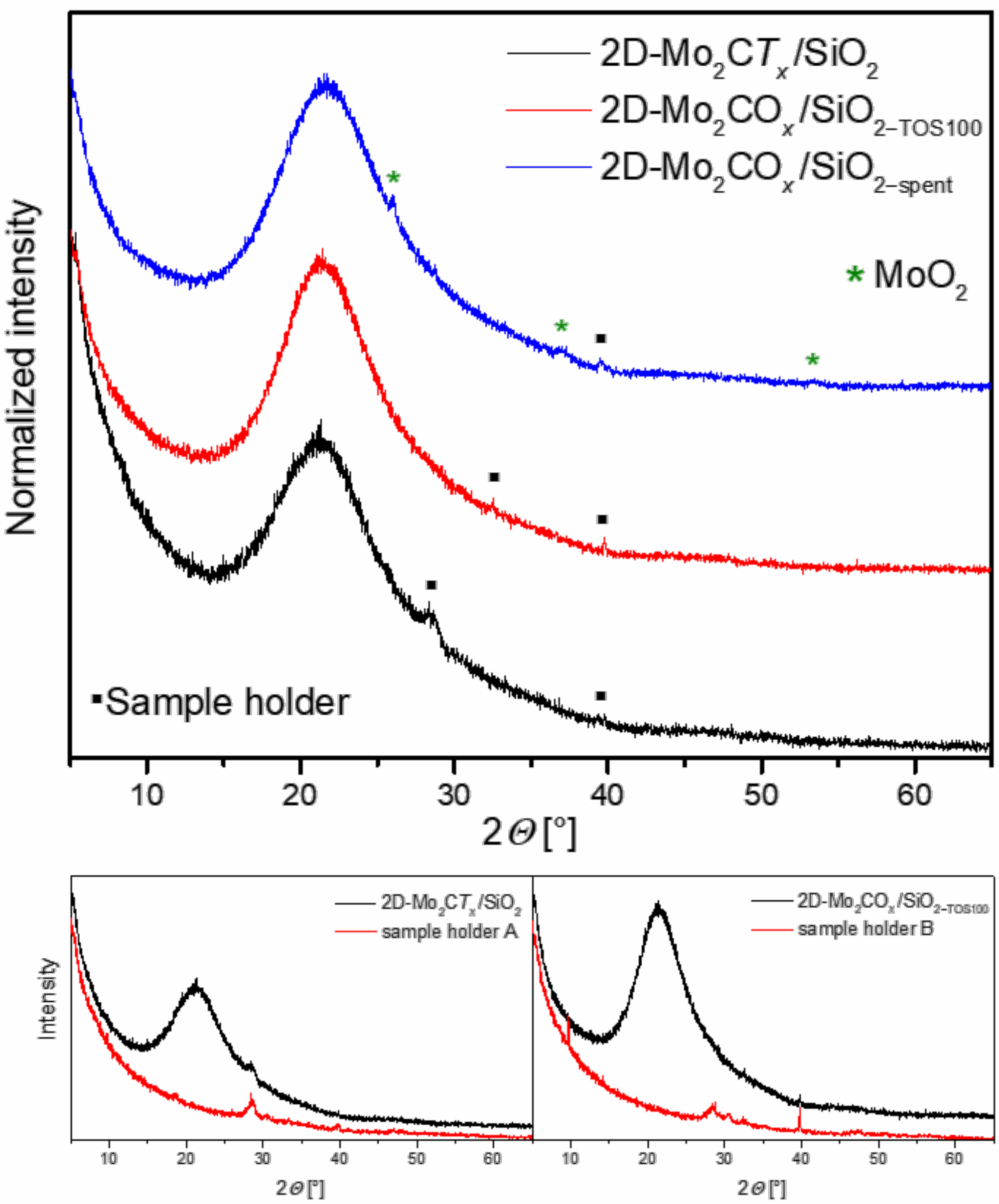

Supplementary Figure 6. XRD pattern of 2D-Mo_2CT_x/SiO_2, 2D-$Mo_2CO_x/SiO_{2\text{-TOS100}}$ and 2D-$Mo_2CO_x/SiO_{2\text{-spent}}$.

XRD pattern of 2D-Mo_2CT_x/SiO_2 and 2D-$Mo_2CO_x/SiO_{2\text{-TOS100}}$ show no presence of crystalline peaks confirming a good dispersion of individual Mo_2CT_x nanosheets and their impressive stability under DRM conditions.

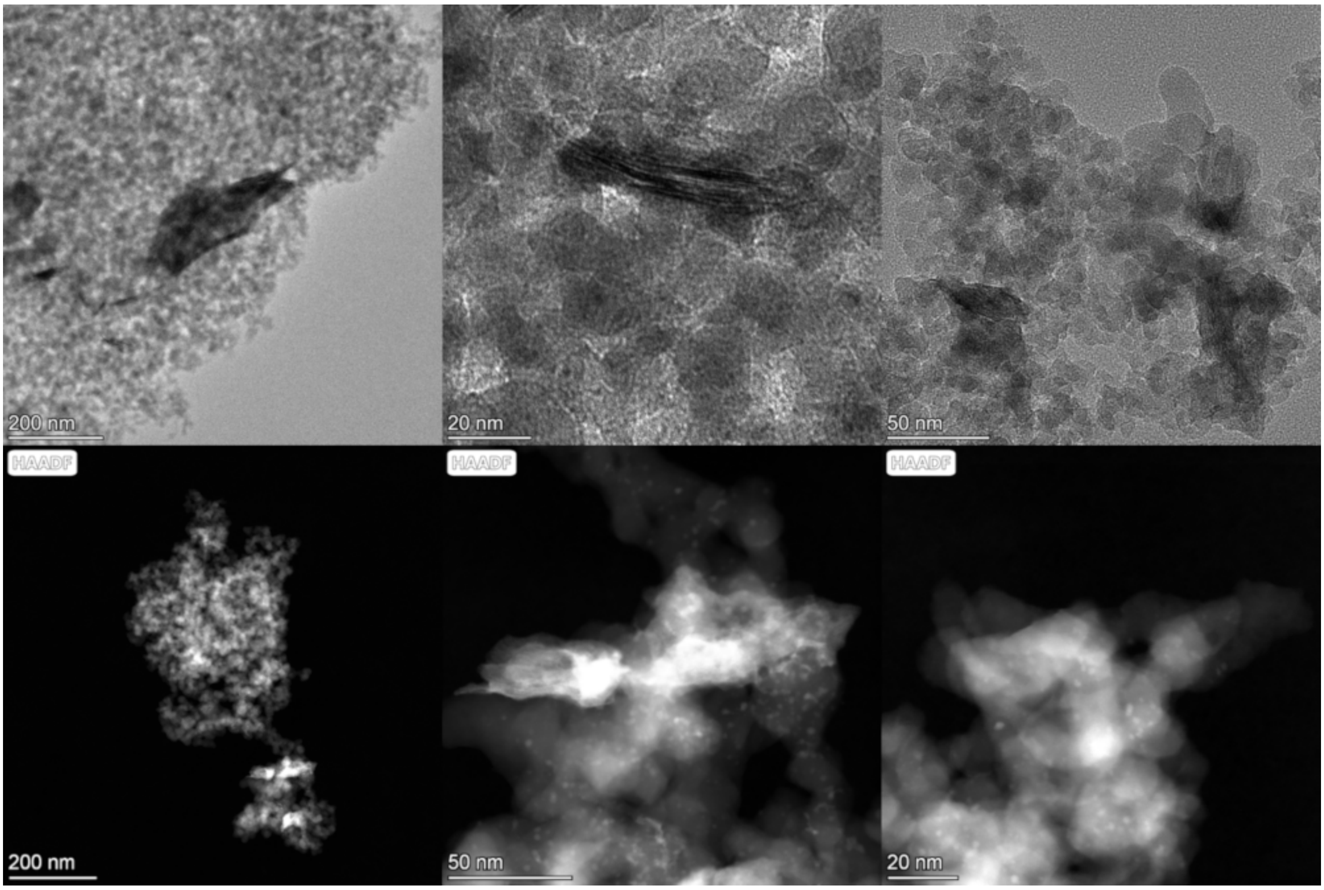

Supplementary Figure 7. TEM images of 2D-Mo_2CT_x/SiO_2 aged in air for ca. 1 month.

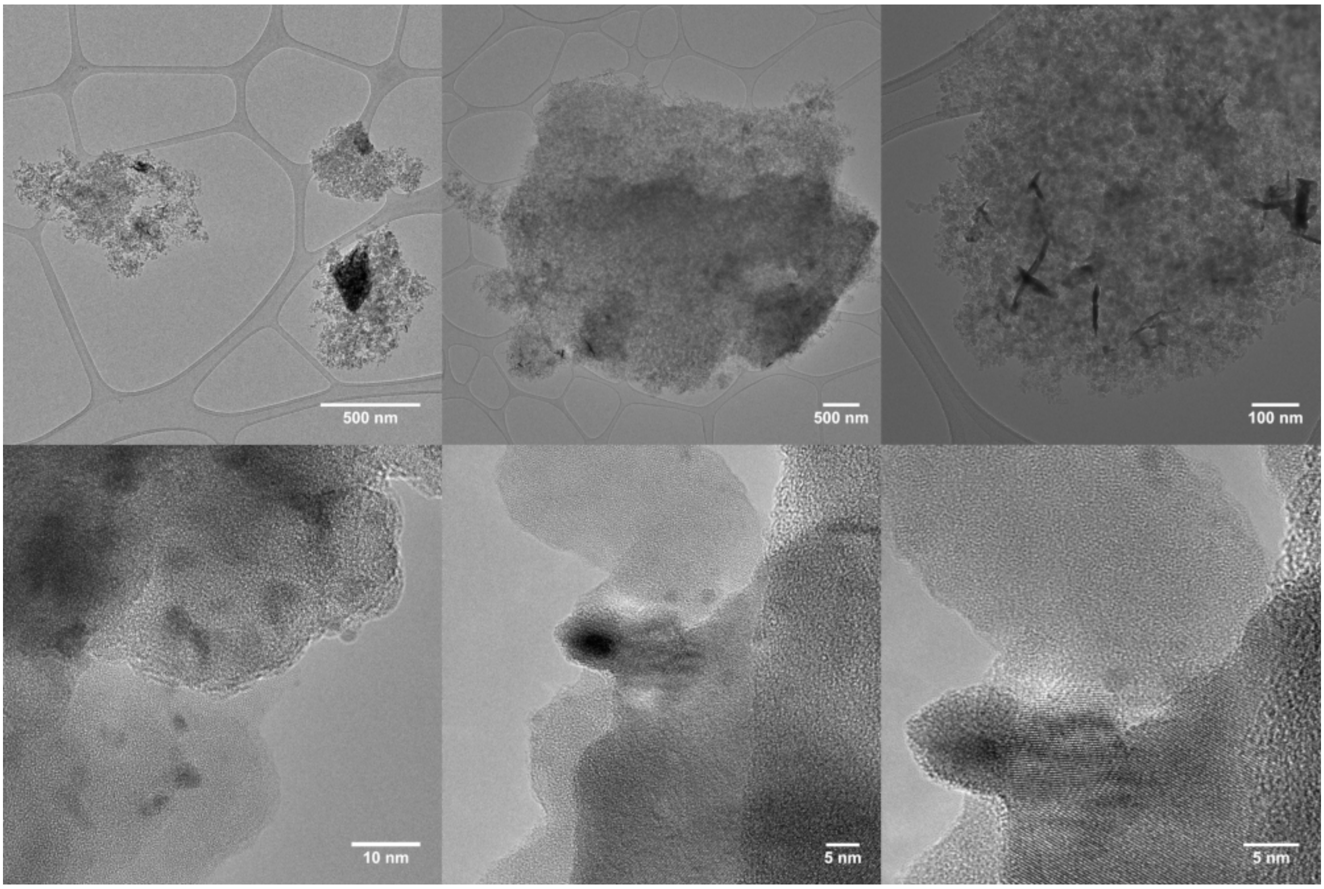

Supplementary Figure 8. TEM images of 2D-Mo_2C/SiO_2.

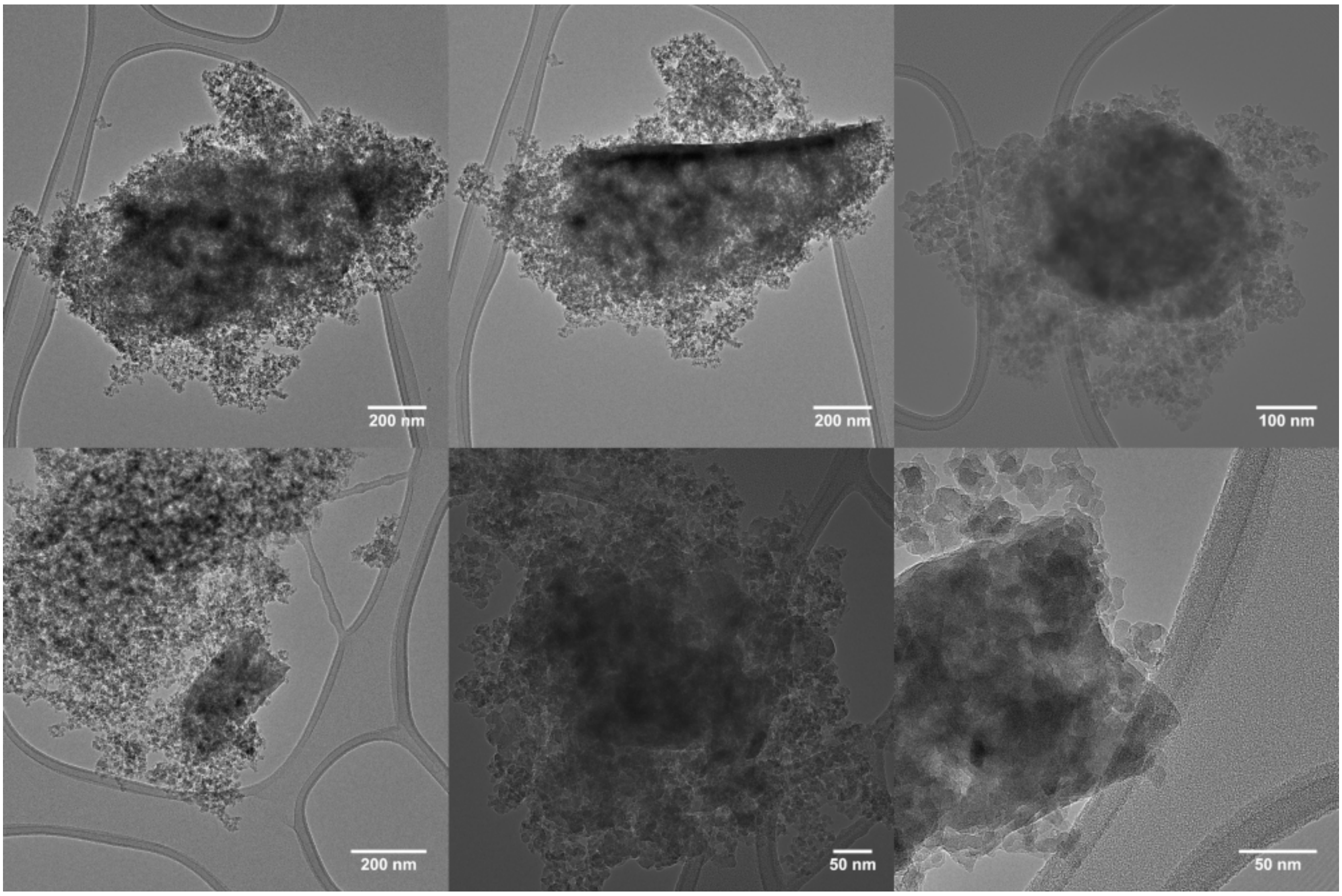

Supplementary Figure 9. TEM images of 2D-Mo_2CO_x/SiO_2.

Note that top left and top middle images represent the same agglomerate of Aerosil 300 particles. A scrolling of 2D-Mo_2CO_x nanosheets was observed after exposure of the specimen on the top left to the higher electron beam doze rate (top middle), confirming that the higher contrast structure on the top left image (and, therefore, on all other TEM images) corresponds to 2D-Mo_2CO_x nanosheets.

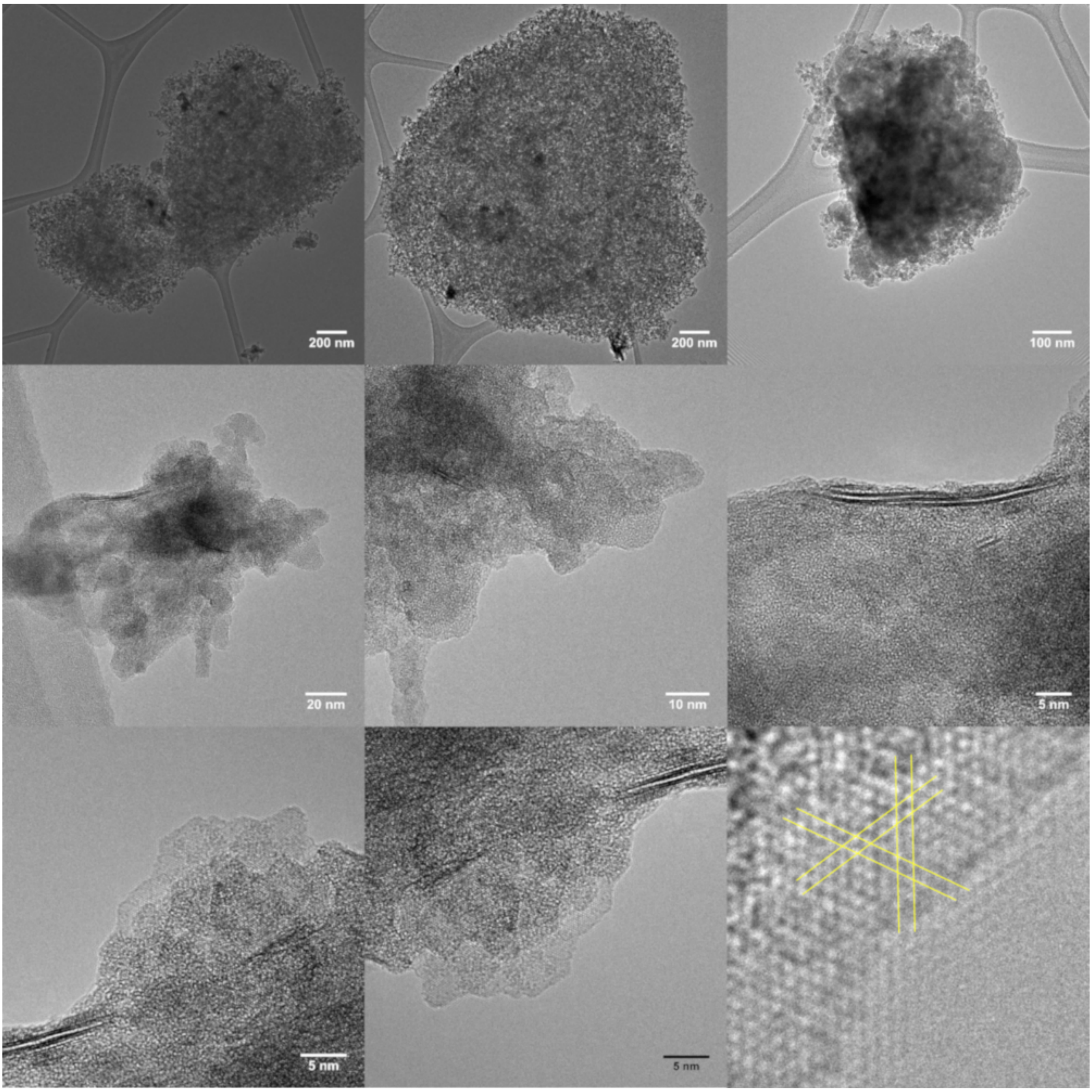

Supplementary Figure 10. TEM images of 2D-$Mo_2CO_x/SiO_{2\text{-regen}}$

This sample was regenerated in CO_2 at 800 °C for 1 h after 100 min of DRM reaction.

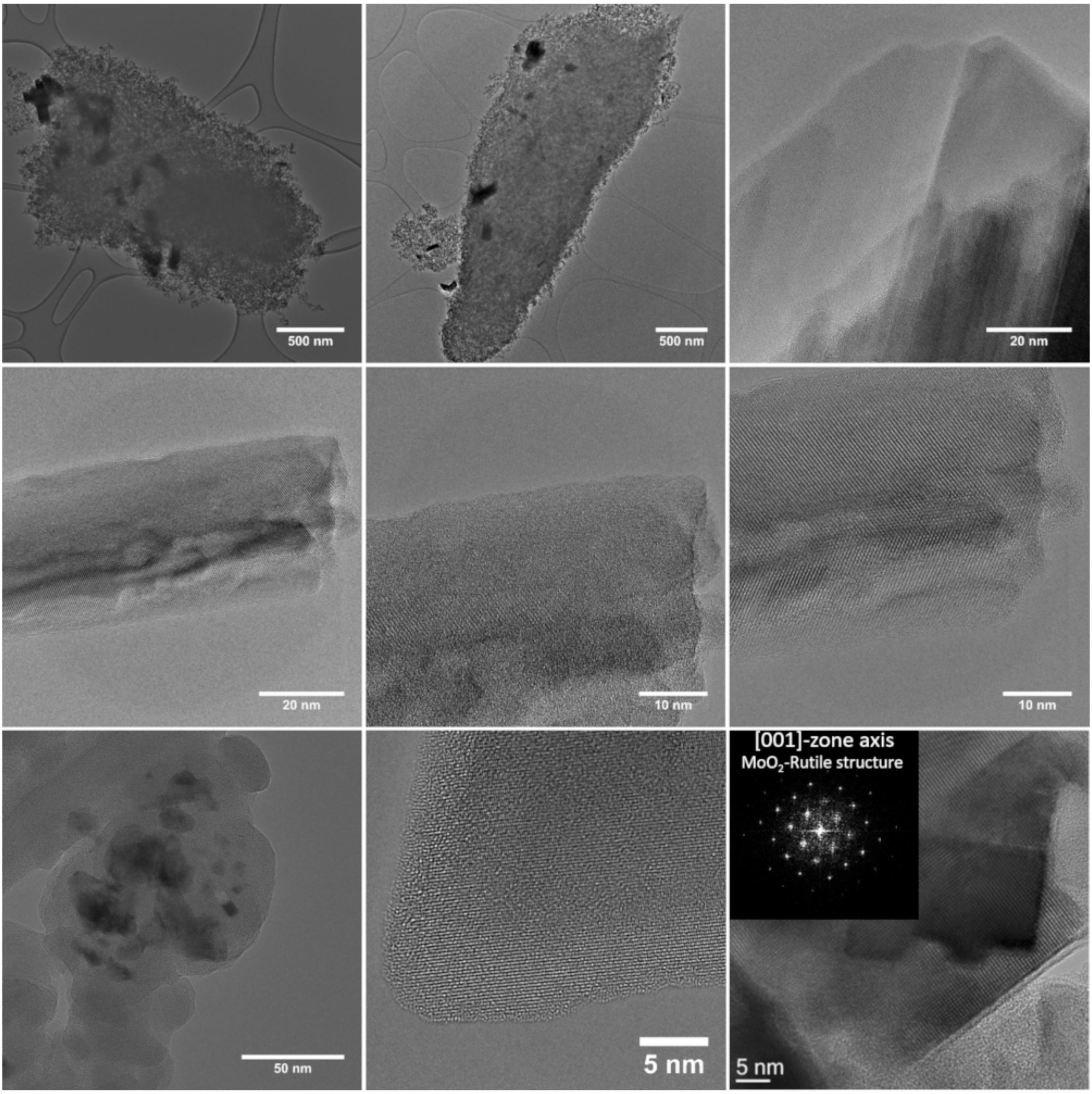

Supplementary Figure 11. TEM images of 2D-$Mo_2CO_x/SiO_{2\text{-spent}}$.

The presence of both 2D nanosheets and MoO_2 particles is observed

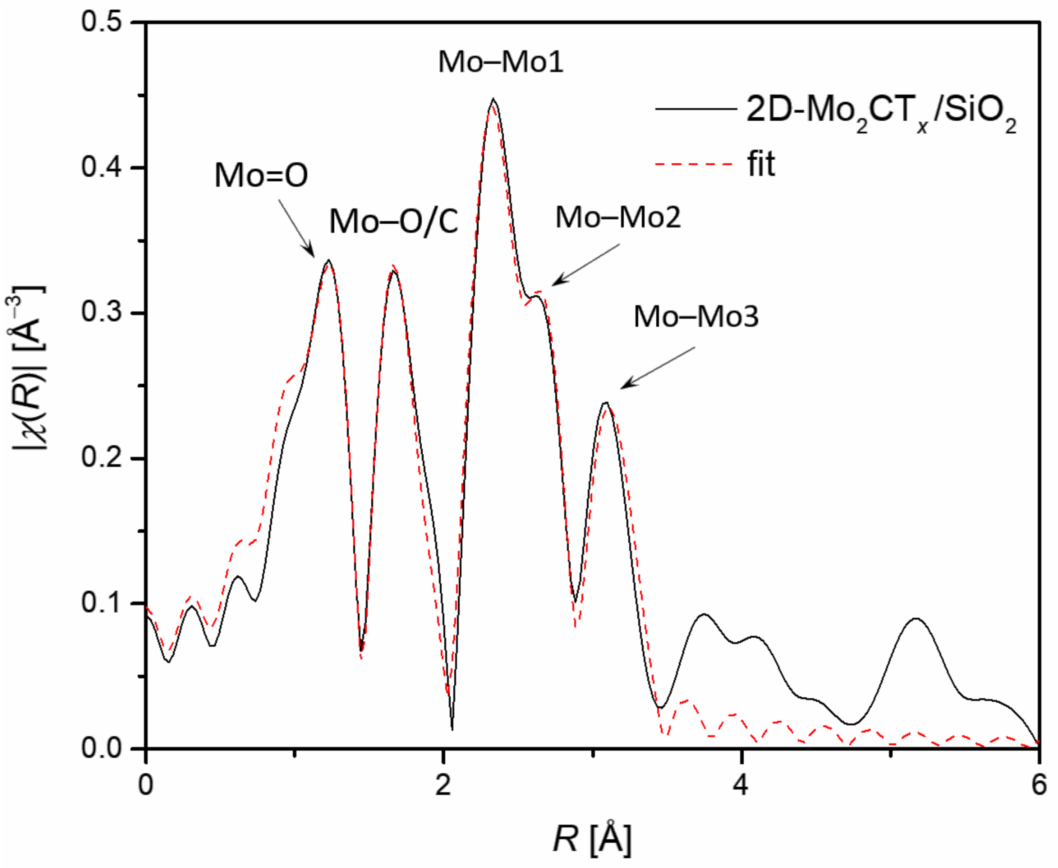

Supplementary Figure 12. EXAFS fitting of 2D-Mo_2CT_x/SiO_2.

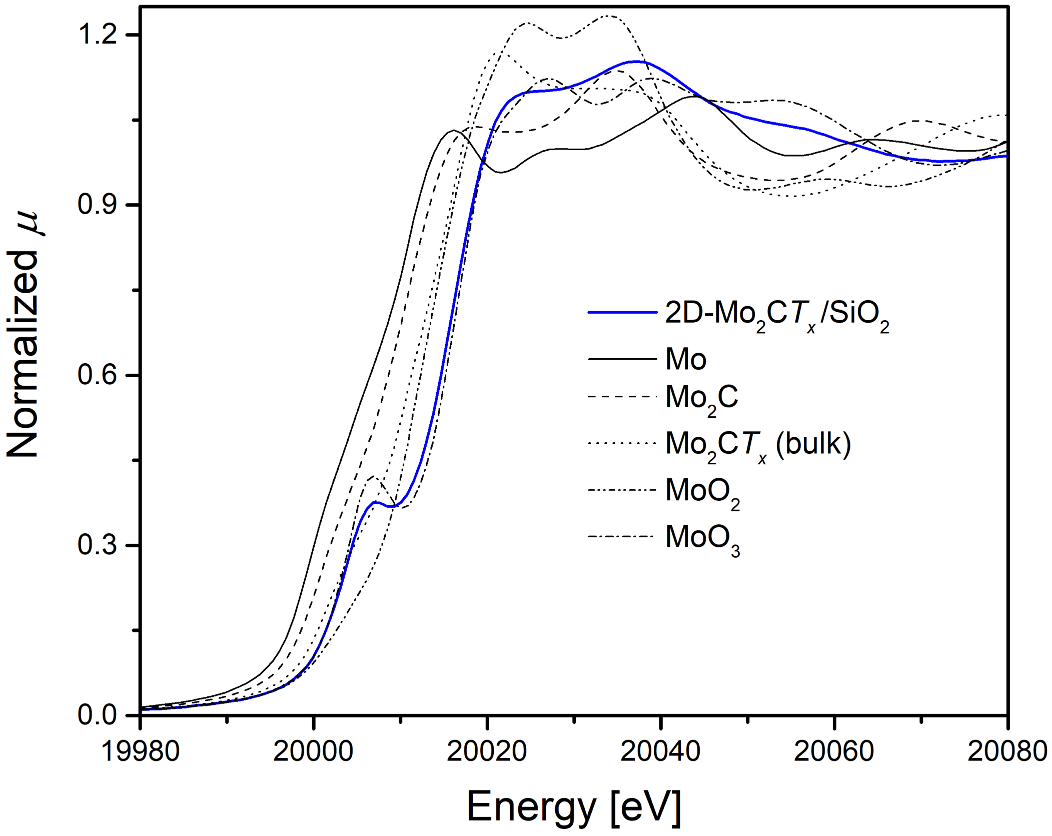

Supplementary Figure 13. XANES spectra of 2D-Mo_2CT_x/SiO_2 and reference materials.

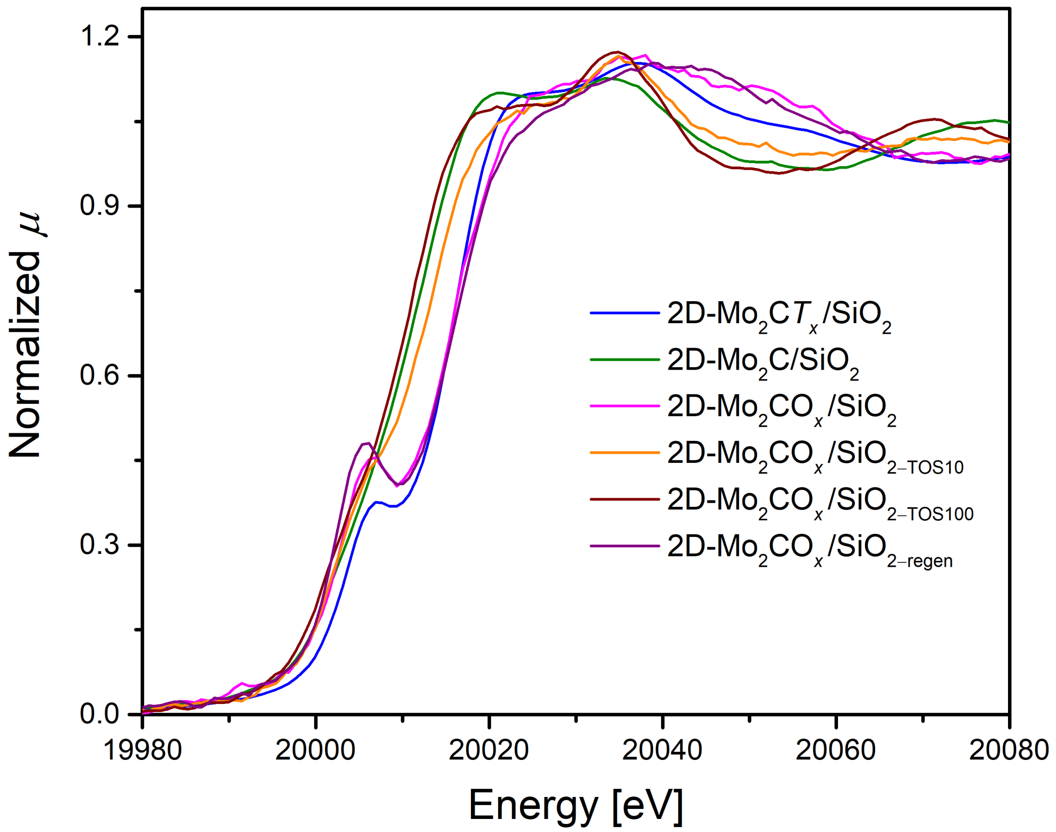

Supplementary Figure 14. XANES spectra of 2D-Mo_2CT_x/SiO_2 after different treatments.

Supplementary Table 1. Mo *K*-edge position and calculated Mo oxidation state for 2D-Mo_2CT_x/SiO_2 treated under different conditions and reference materials.

Sample	Mo *K*-edge position (eV)	Mo oxidation state
2D-Mo_2CT_x/SiO_2	20015.8	5.5
2D-Mo_2C/SiO_2	20000.8	0.2
2D-Mo_2CO_x/SiO_2	20015.7	5.5
2D-$Mo_2CO_x/SiO_{2\text{–TOS10}}$	20011.5	4.0
2D-$Mo_2CO_x/SiO_{2\text{–TOS100}}$	20009.0	3.0
2D-$Mo_2CO_x/SiO_{2\text{–regen}}$	20015.9	5.5
Mo	20000.0	0
β-Mo_2C	20000.7	0.2
MoO_2	20011.4	4
MoO_3	20017.0	6
Mo_2CT_x	20010.9	3.8

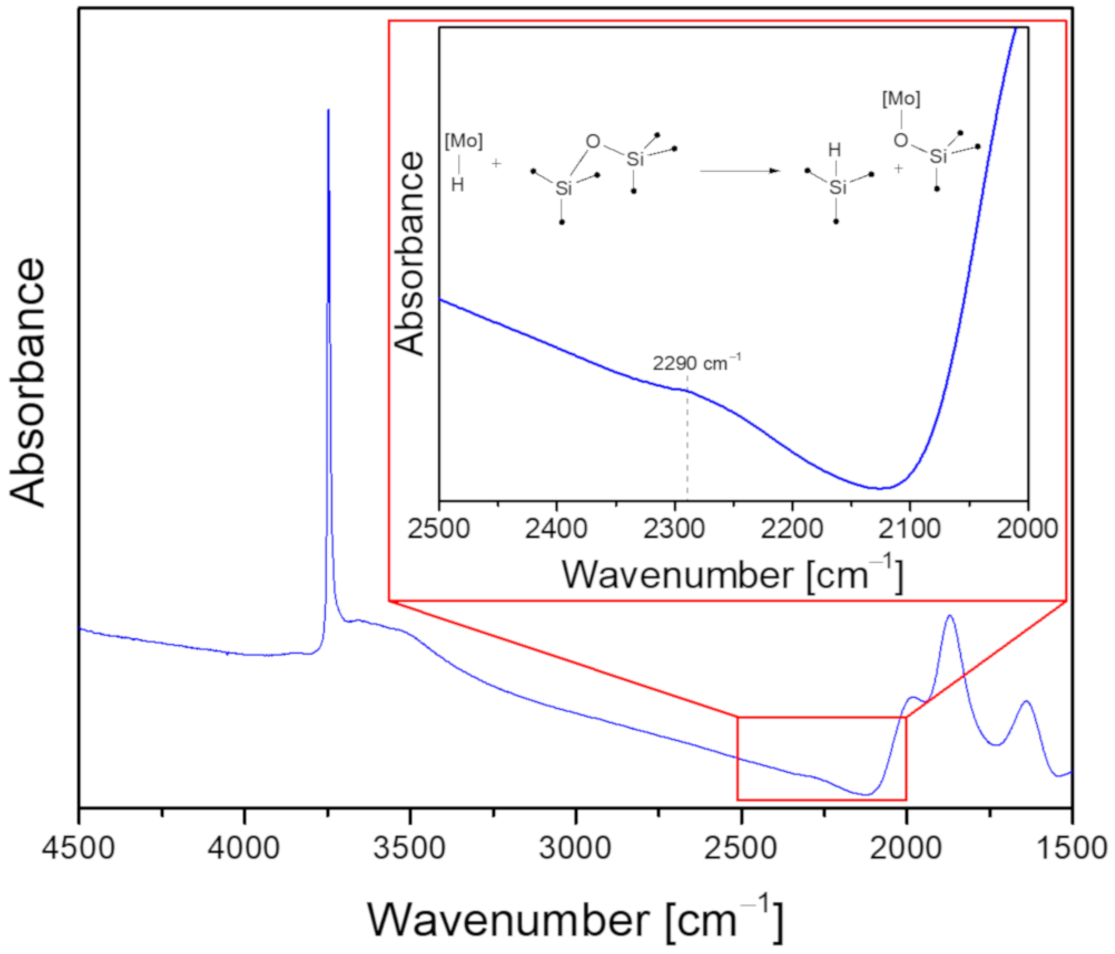

Supplementary Figure 15. IR spectrum of 2D-Mo_2C/SiO_2 with a zoomed in area of a [≡Si−H] region.

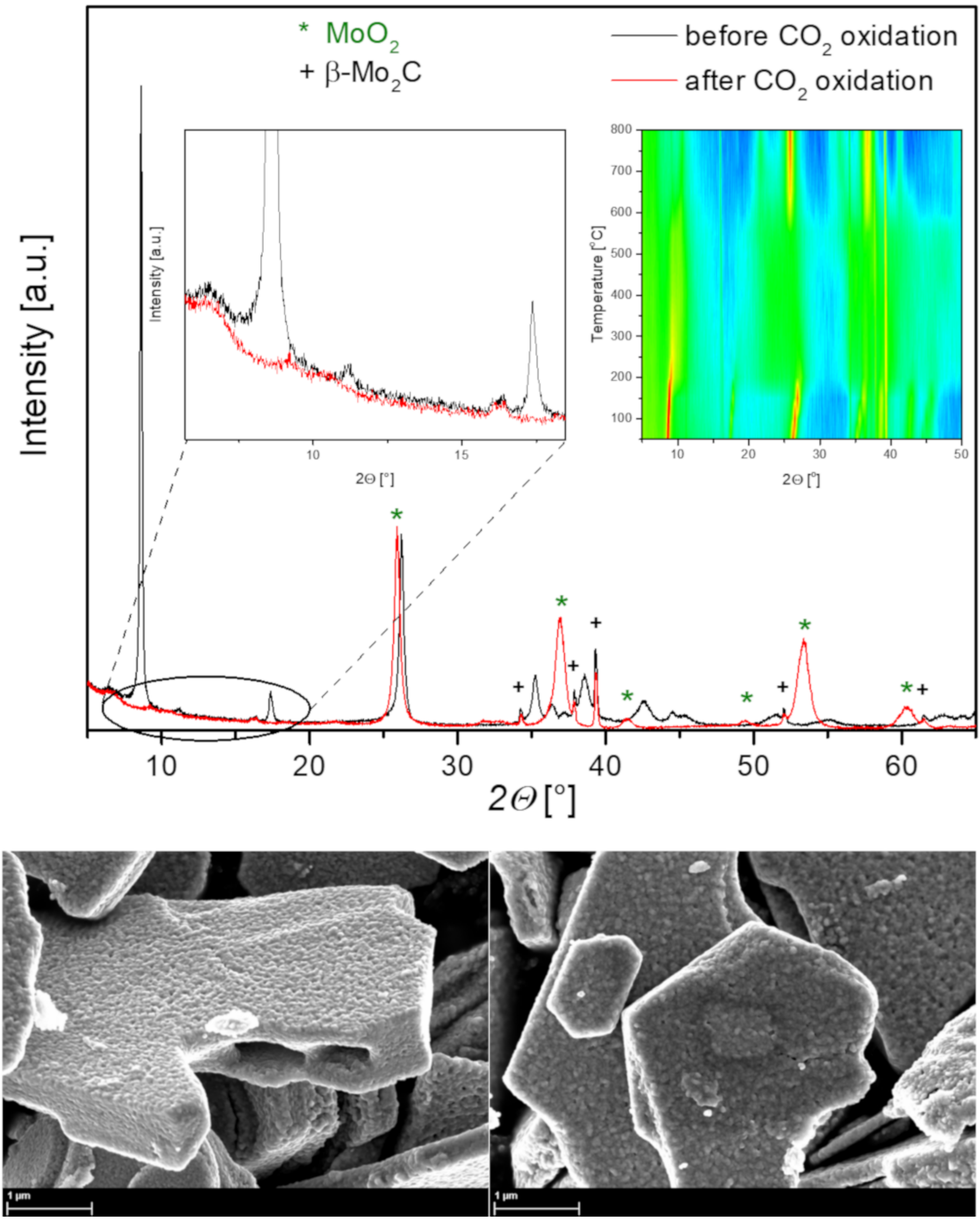

Supplementary Figure 16. Top: XRD pattern of *m*-Mo_2CT_x before (black) and after (red) oxidation in CO_2 at 800 °C. Inset: In situ XRD pattern of bulk Mo_2CT_x oxidation in CO_2 (20 vol. % CO_2 in N_2, XRD pattern every 50 °C). Bottom: SEM images of Mo_2CT_x annealed at 800 °C in pure CO_2.

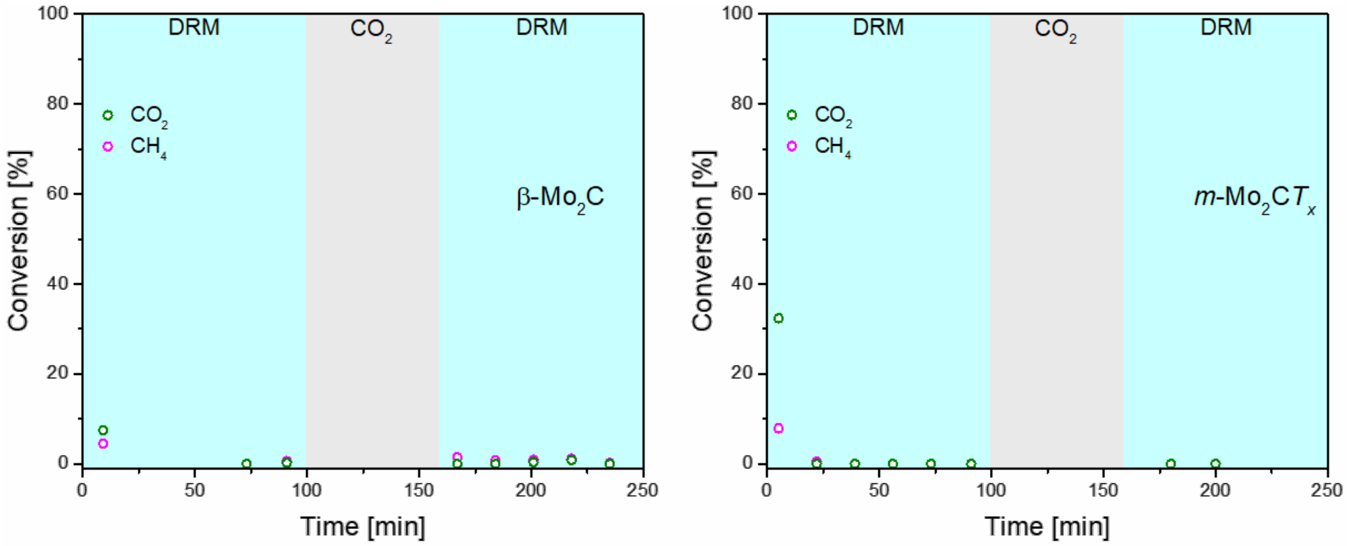

Supplementary Figure 17. Catalytic performance of β-Mo_2C (left) and *m*-Mo_2CT_x (right) in DRM.

Reaction conditions: 800 °C, 1 bar, CH_4:CO_2= 1:1, total flow rate of 20 mL min^{-1}, 0.1 and 0.03 g of catalyst, respectively.

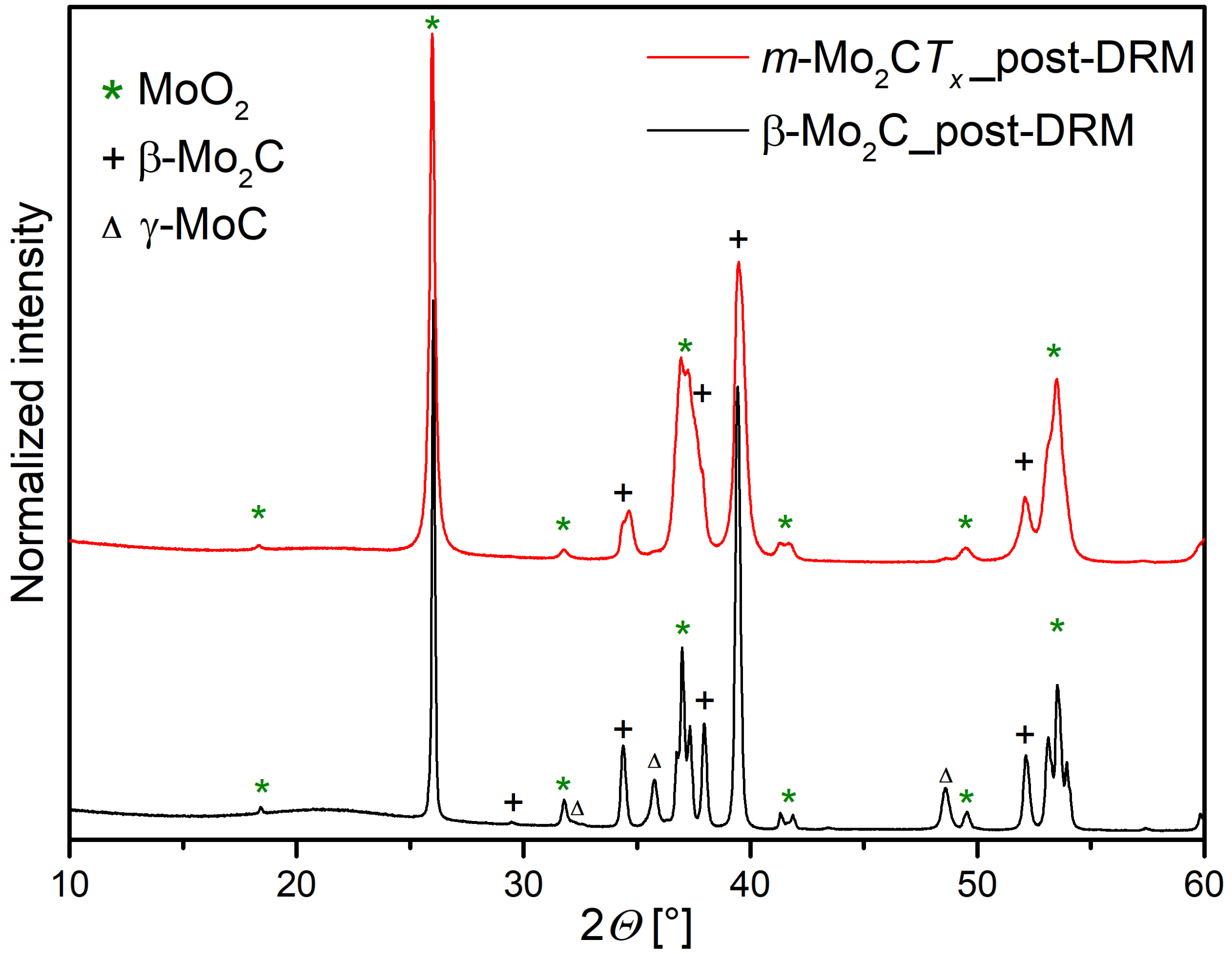

Supplementary Figure 18. XRD patterns of β-Mo_2C and *m*-Mo_2CT_x after the DRM catalytic test.

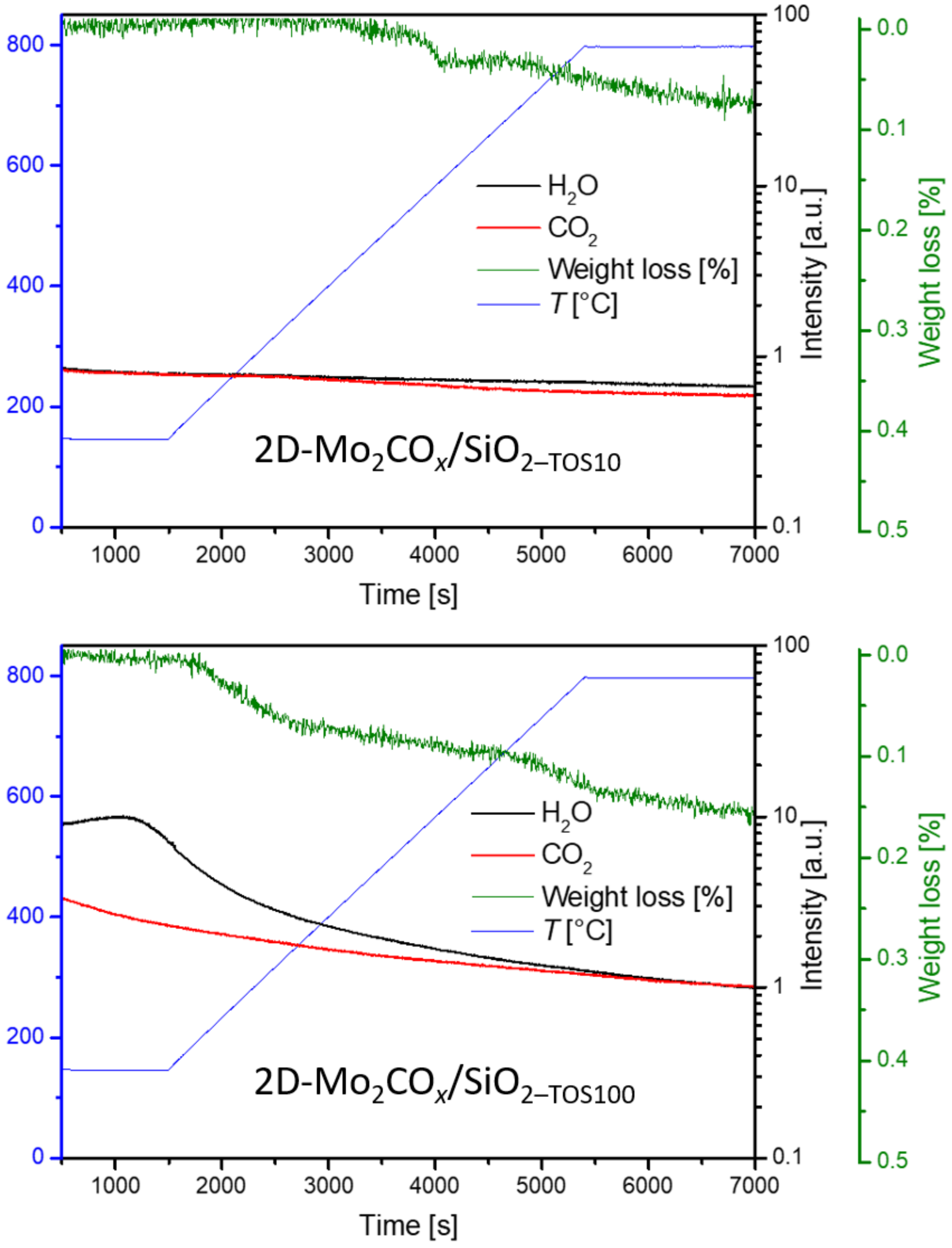

Supplementary Figure 19. A temperature programed oxidation (TPO) of 2D-$Mo_2CO_x/SiO_{2\text{-TOS10}}$ (top) and 2D-$Mo_2CO_x/SiO_{2\text{-TOS100}}$ (bottom) in a TGA followed by MS.

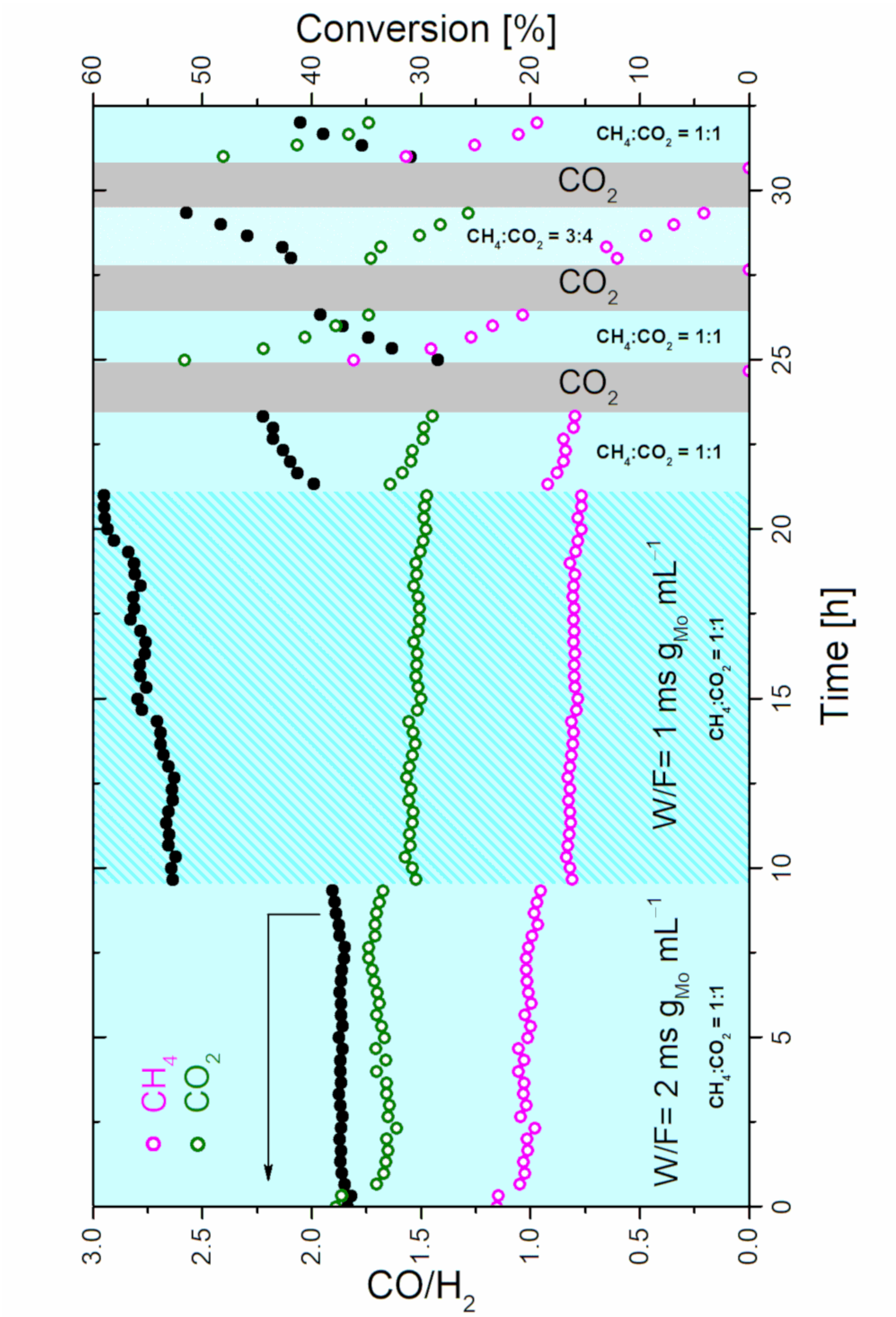

Supplementary Figure 20. DRM stability test of 2D-Mo_2CO_x/SiO_2 with several regeneration/deactivation cycles.

After the last data point, the sample was recovered for characterization (2D-$Mo_2CO_x/SiO_{2\text{-spent}}$)

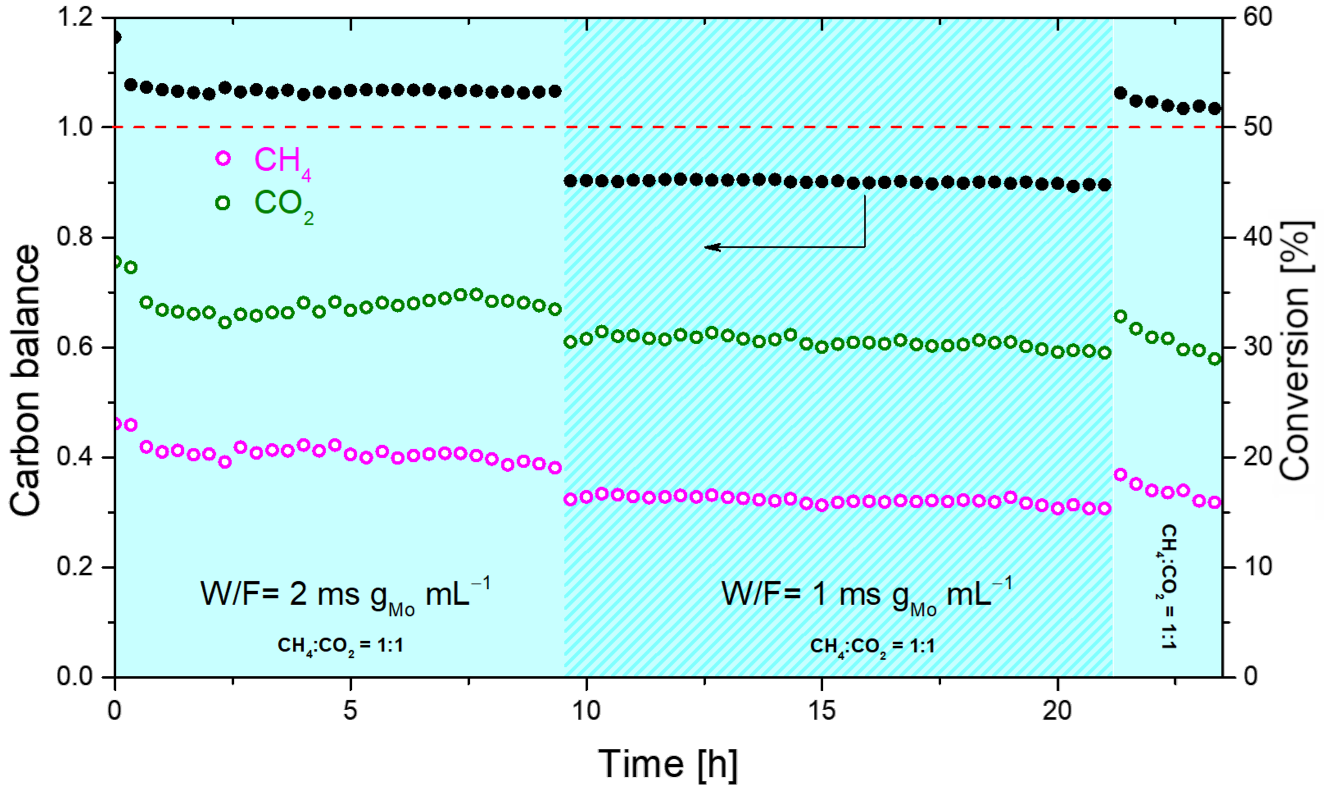

Supplementary Figure 21. DRM stability test of 2D-Mo_2CO_x/SiO_2 (CH_4 and CO_2 conversion, %) with the corresponding carbon balance determined by GC.

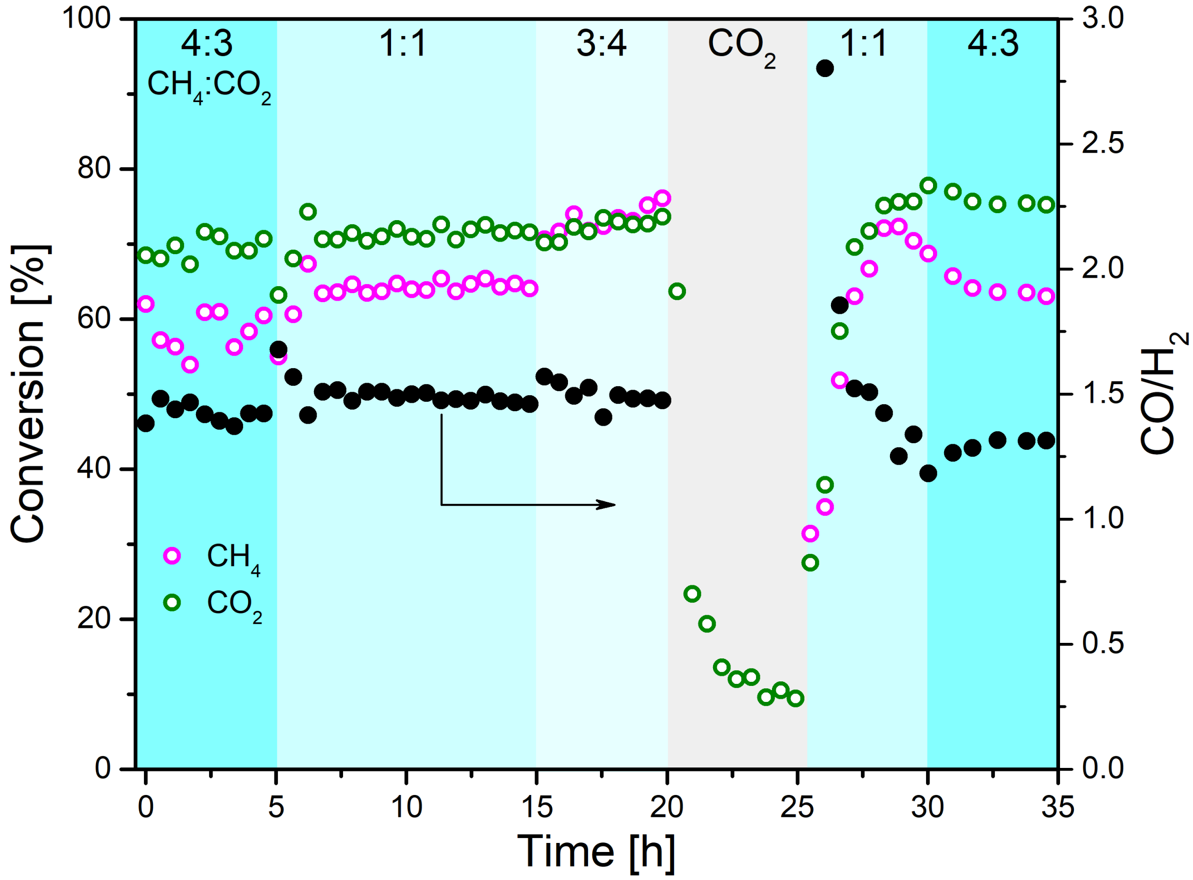

Supplementary Figure 22. Catalytic performance of 2D-Mo_2CT_x/SiO_2 in DRM at elevated pressure. Reaction conditions: 800 °C, 8 bar, CH_4:CO_2= 1:1, total flow rate of 10 mL min^{-1}, 0.1 g of catalyst.

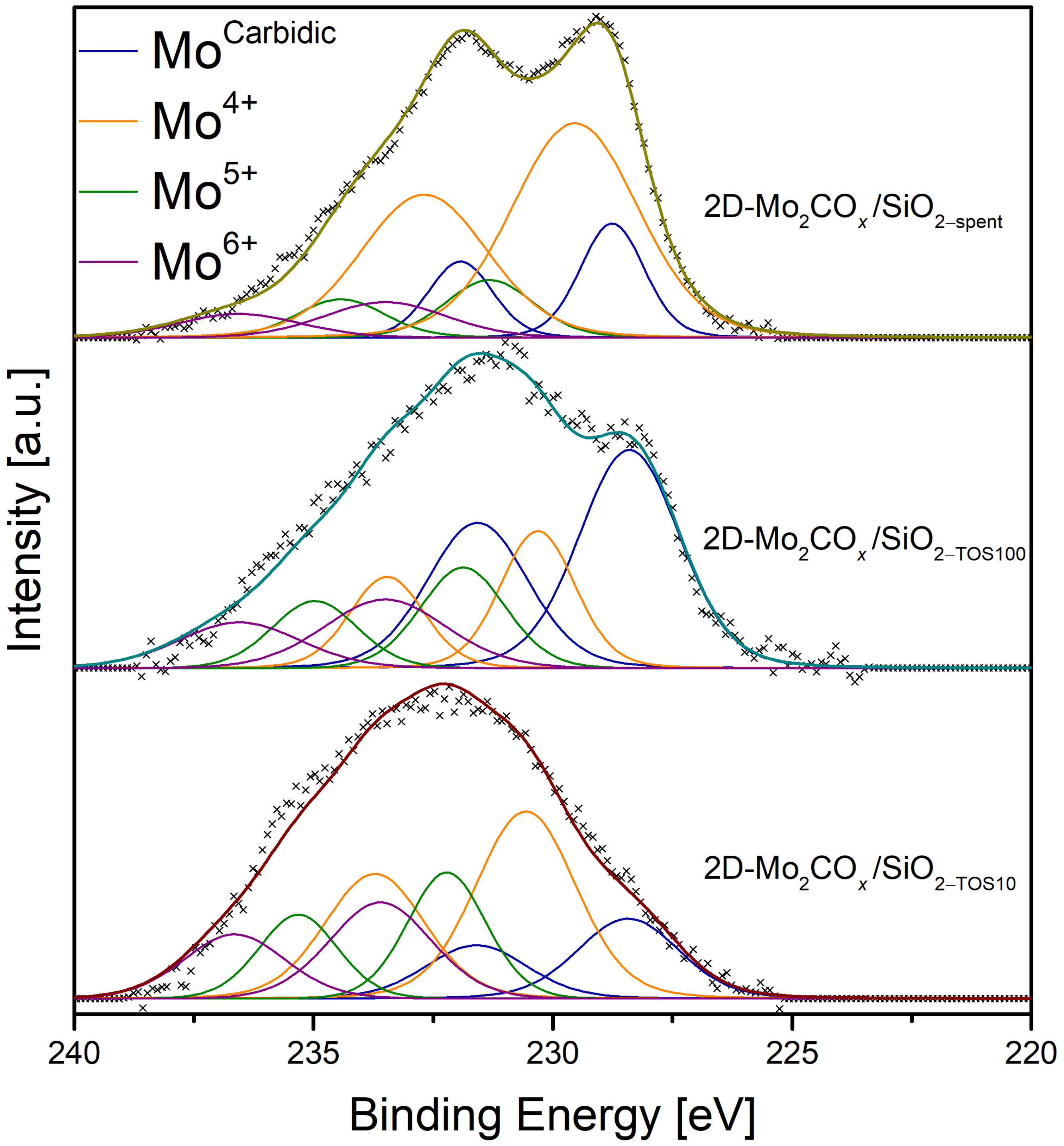

Supplementary Figure 23. Deconvolution of ex situ XPS data of the Mo *3d* core levels of 2D-$Mo_2CO_x/SiO_{2\text{-TOS10}}$, 2D-$Mo_2CO_x/SiO_{2\text{-TOS100}}$ and 2D-$Mo_2CO_x/SiO_{2\text{-spent}}$.

Supplementary Table 2. XPS fitting parameters for Mo_2CT_x, 2D-Mo_2CT_x/SiO_2, 2D-Mo_2C/SiO_2, 2D-Mo_2CO_x/SiO_2, 2D-Mo_2CO_x/SiO_2-DRM, β-Mo_2C and MoO_3.

State	Mo $3d_{5/2}$ B.E. [eV]	ΔB.E. [eV]	FWHM [eV]	Peak shape	Amount [%]
			Mo_2CT_x		
Mo^{4+}	229.6	3.15	1.31	LA*	72
Mo^{5+}	232.6	3.06	2.62	GL#	28
			2D-Mo_2CT_x/SiO_2		
Mo^{4+}	229.8	3.15	1.45	LA	9
Mo^{5+}	232.1	3.10	2.70	GL	40
Mo^{6+}	233.6	3.05	2.50	GL	51
			2D-Mo_2C/SiO_2		
Carbidic	228.8	3.17	2.09	LA	53
Mo^{4+}	230.2	3.15	2.15	LA	29
Mo^{6+}	233.1	3.05	3.00	GL	18
			2D-Mo_2CO_x/SiO_2		
Mo^0	228.0	3.17	2.00	LA	23
Mo^{4+}	230.1	3.10	2.24	LA	40
Mo^{5+}	232.1	3.10	1.86	GL	13
Mo^{6+}	233.5	3.05	3.00	GL	24
			2D-$Mo_2CO_x/SiO_{2-TOS10}$		
Carbidic	228.4	3.17	2.50	LA	18
Mo^{4+}	230.6	3.15	2.46	LA	41
Mo^{5+}	232.2	3.10	1.93	GL	21
Mo^{6+}	233.6	3.05	2.50	GL	20
			2D-$Mo_2CO_x/SiO_{2-TOS100}$		
Carbidic	228.4	3.17	2.50	LA	45
Mo^{4+}	230.3	3.15	1.84	LA	21
Mo^{5+}	231.9	3.10	2.10	GL	17
Mo^{6+}	233.5	3.05	2.99	GL	17
			2D-$Mo_2CO_x/SiO_{2-spent}$		
Carbidic	228.8	3.17	1.58	LA	17
Mo^{4+}	229.5	3.15	3.11	LA	62
Mo^{5+}	231.3	3.10	2.17	GL	11
Mo^{6+}	233.5	3.05	3.00	GL	10
			β-Mo_2C		
Carbidic	228.5	3.17	1.26	LA	100
			MoO_3		
Mo^{5+}	232.2	3.05	2.24	GL	13
Mo^{6+}	233.3	3.13	1.41	GL	87

* LA- Lorentzian Asymmetric line shape, # GL- Gaussian/Lorentzian line shape.

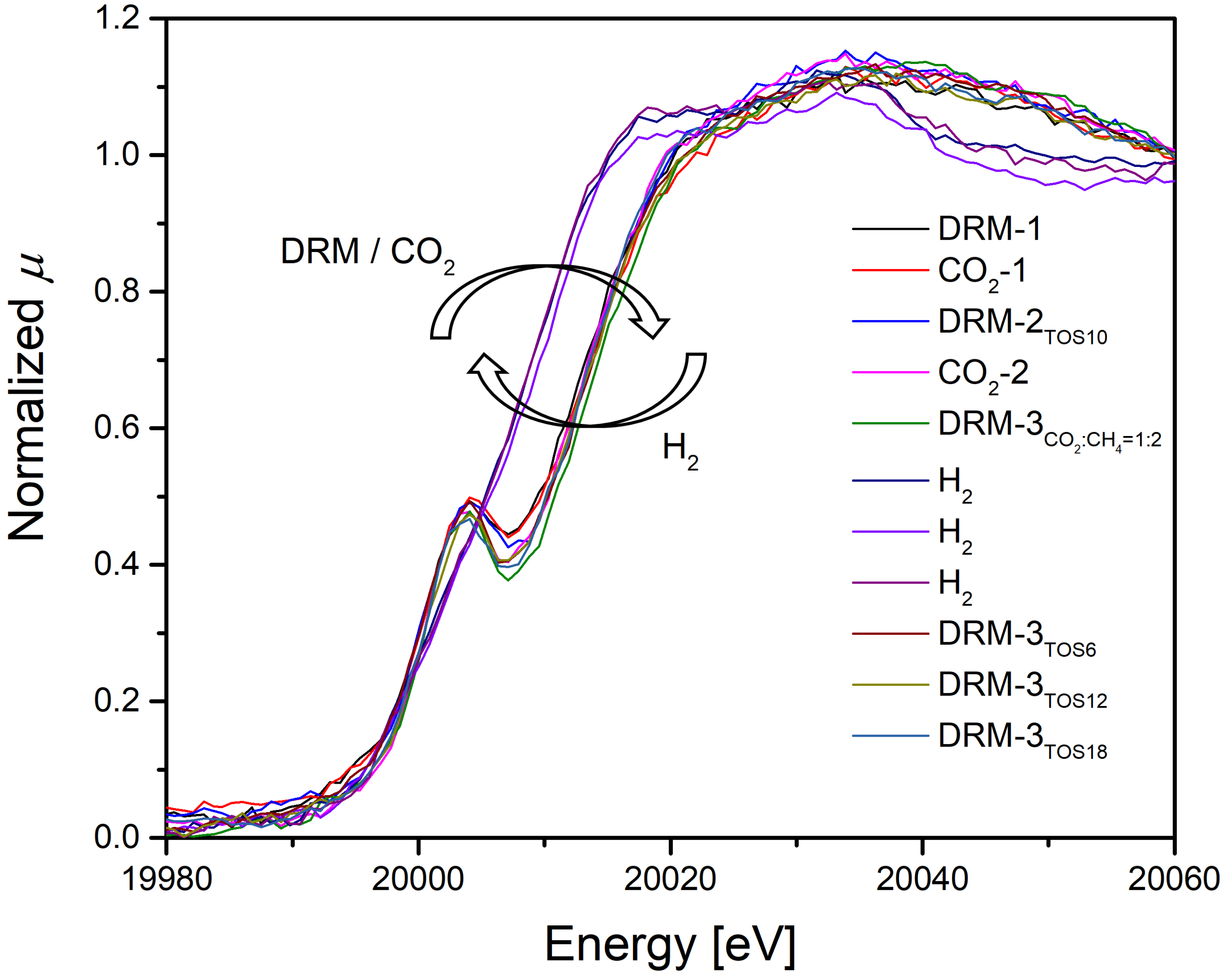

Supplementary Figure 24. XANES spectra of 2D-Mo_2CT_x derived materials under different conditions representing a complete reversibility between 2D-Mo_2C/SiO_2 and 2D-Mo_2CO_x/SiO_2.

Supplementary Table 3. Comparison of the DRM activity of 2D-Mo_2CO_x/SiO_2 with literature data for Mo-based catalysts.

	Catalyst	T °C	SV mL g^{-1} h^{-1}	P bar	$X(CH_4)$ %	Activity mol_{CH4} mol_{Mo}^{-1} s^{-1}	H_2:CO	Ref.
1	30 wt.% Mo_2C/CNT	800	20400	1	10	0.003	–	[1]
2	6.3% Mo_2C/Al_2O_3	800	20000 h^{-1}	1	25.9	–	0.73	[2]
3	5%Ni- 4.2%Mo_2C/Al_2O_3	800	20000 h^{-1}	1	78.6	–	0.89	[2]
4	Ni/Mo_2C (1:9)	800	6000	1	80	0.003 and 0.028‡	–	[3]
5	β-Mo_2C	800	6000	1	<1	0	–	[3]
6	4.4%Ni-14.6%Mo_2C/La_2O_3	800	12000	1	66	0.033 and 0.067‡	–	[4]
7	Ni-Mo_2C (1:2)	800	12000	1	80	0.009 and 0.02‡	–	[4]
8	Mo_2C	800	6000	1	7	0.0002	<0.1	[5]
9	Ni-Mo_2C (1:1)	800	6000	1	82	0.005 and 0.005‡	0.55	[5]
10	2D-Mo_2CO_x/SiO_2	800	12000*	1	80	0.42	0.85#	This work
11	2D-Mo_2CO_x/SiO_2	800	12000*	8	64	0.34	0.7	This work
12	m-Mo_2CT_x	800	40000	1	8	0.002	0.33	This work
13	β-Mo_2C	800	12000	1	4.5	0.0003	0.3	This work
14	30 wt.% Mo_2C/CNT	850	18000	1	85	0.03	–	[1]
15	β-Mo_2C	850	18000	1	<1	0	–	[1]
16	α-MoC_{1-x}	850	6000	1	87	0.002	–	[6]
17	β-Mo_2C	850	6000	1	20	0.0005	–	[6]
18	18.3% Mo_2C/SiO_2	850	2600 h^{-1}	8	91	–	0.95	[7]
19	β-Mo_2C	850	2800 h^{-1}	8.3	62.5	–	0.78	[8]
20	5% Mo_2C/Al_2O_3	850	2800 h^{-1}	8.3	65.2	–	0.81	[8]

* Per "g" of catalyst. 0.48% Mo loading
‡ Activity per mole of Ni: mol_{CH4} mol_{Ni}^{-1} s^{-1}
# Corresponds to the catalytic data presented in Figure 2 in the main text (entry 10), as well as in Supplementary Figure 22 (entry 11).

Supplementary Table 4. Total and areal CO capacity as well as the specific (Mo_2C) surface area of 2D-Mo_2C/SiO_2 and β-Mo_2C.

	2D-Mo_2C/SiO_2	β-Mo_2C
CO adsorbed, 1-st isotherm, μmol g_{cat}^{-1}	0.7	0.52
CO adsorbed, 2-nd isotherm, μmol g_{cat}^{-1}	0.4	0.03
CO capacity, μmol g_{cat}^{-1}	0.3	0.5
Specific Mo_2C surface area, m^2 g_{cat}^{-1}	1.34*	1-2
Areal CO capacity, μmol m^{-2}	0.22	0.25-0.5

* Estimated based on the crystal structure of Mo_2CT_x, see general experimental for details.

Supplementary Methods

Supplementary Computational Details

Structure of 2D-Mo_2C under CO_2 atmosphere

We evaluated different compositions of species on the 2D-Mo_2C slab and the resulting stabilities at 800 °C. For the reaction of one CO_2 molecule with the 2D-Mo_2C slab, the summary of reactions evaluated together with the resulting energetics is shown below.

$CO_{2(g)} \rightarrow CO_{(g)} + O^*$ $\Delta G = -77$ kJ mol^{-1}
$CO_{2(g)} \rightarrow CO^* + O^*$ $\Delta G = -105$ kJ mol^{-1}
$CO_{2(g)} \rightarrow C^* + 2O^*$ $\Delta G = -187$ kJ mol^{-1}
$2CO_{2(g)} \rightarrow 2C^* + 4O^*$ $\Delta G = -228$ kJ mol^{-1}
$3CO_{2(g)} \rightarrow 3C^* + 6O^*$ $\Delta G = -229$ kJ mol^{-1}
$9CO_{2(g)} \rightarrow 9CO_{(g)} + 9O^*$ $\Delta G = -464$ kJ mol^{-1}
$18CO_{2(g)} \rightarrow 18CO_{(g)} + 18O^*$ $\Delta G = -928$ kJ mol^{-1}

The Gibbs energy of cleaving one CO_2 molecule, releasing one CO molecule in the gas phase and adsorbing one oxygen atom onto 2D-Mo_2C is exergonic by –77 kJ mol^{-1}. If CO* remains adsorbed, than the Gibbs energy is equal to –105 kJ mol^{-1}. This suggests that further CO* dissociation should be favored. Indeed, the formation of one C* and 2O* species is exergonic by –187 kJ mol^{-1}. If three CO_2 molecules are fully dissociated forming 3C* and 6O*, the Gibbs energies converges to a value equal to –229 kJ mol^{-1}. Nevertheless, the release of CO(g) together with the adsorption of atomic O* has less entropic penalty than the adsorption of all the species on the surface. For instance, if 9CO_2 molecules react on the top of the 2D-Mo_2C surface giving raise to 1 O ML on the top surface, this configuration is significantly more stable than all the previous ones, taking place in a highly exergonic process by –464 kJ mol^{-1}. Further addition of oxygen on the top surface by surface releasing another CO molecule in the gas phase or the formation of subsurface oxygen is an endergonic process. The adsorption of 9 additional oxygen atoms on the bottom layer of the Mo_2C slab, with the release of nine additional CO molecules has exactly the same energetics than the same reaction on the top layer, i.e. adsorbing a total of 18 oxygen atoms (9 per Mo layer) has an overall Gibbs energy exergonic by –928 kJ mol^{-1}. Thus, in atmosphere of pure CO_2 at 800 °C, 1 O ML is expected per Mo atom on both the top and the bottom layer, in agreement with the experimental data. Since the adsorption on each side of the surface is very similar for next steps, we considered the surface with 1 O ML on one side of the slab.

ΔG of reaction as a function of the number of CO_2 molecules that react on the Mo_2C slab:

17 CO_2(g) → 17 CO(g) + O* $\Delta G = -887$ kJ mol^{-1}, average energy = –52 kJ mol^{-1}
18 CO_2(g) → 18 CO(g) + O* $\Delta G = -928$ kJ mol^{-1}, average energy = –52 kJ mol^{-1}
19 CO_2(g) → 19 CO(g) + O* $\Delta G = -903$ kJ mol^{-1}, average energy = –48 kJ mol^{-1}

Structure of the CO_2-treated 2D-Mo_2C surface under the DRM reaction conditions

As a starting point, we took the 2D-Mo_2C surface covered with 1 O ML on the top layer. Then, we evaluated possible reduction of the oxygen adsorbed on such 2D-Mo_2C surface since under DRM, the atmosphere is significantly more reducing than pure CO_2 (the latter atmosphere is oxidizing). We evaluated the reaction of the adsorbed oxygen with methane to form CO and H_2, and we found that it is thermodynamically favorable to reduce the coverage of oxygen of the Mo_2C surface previously treated with CO_2.

$$CH_4 + O^* \rightarrow CO + 2H_2 \qquad \Delta G = -30 \text{ kJ mol}^{-1}$$
$$2CH_4 + 2O^* \rightarrow 2CO + 4H_2 \qquad \Delta G = -18 \text{ kJ mol}^{-1}$$
$$3CH_4 + 3O^* \rightarrow 3CO + 6H_2 \qquad \Delta G = 0 \text{ kJ mol}^{-1}$$

The results show that reducing the oxygen coverage is exergonic for one and two oxygen atoms and isoergonic for three oxygens. When evaluating the DRM reaction, we considered the surface with lower oxygen coverage than 1 O ML and to ease convergence, we selected 2D-Mo_2C-0.67 O ML coverage.

Dry Reforming of Methane

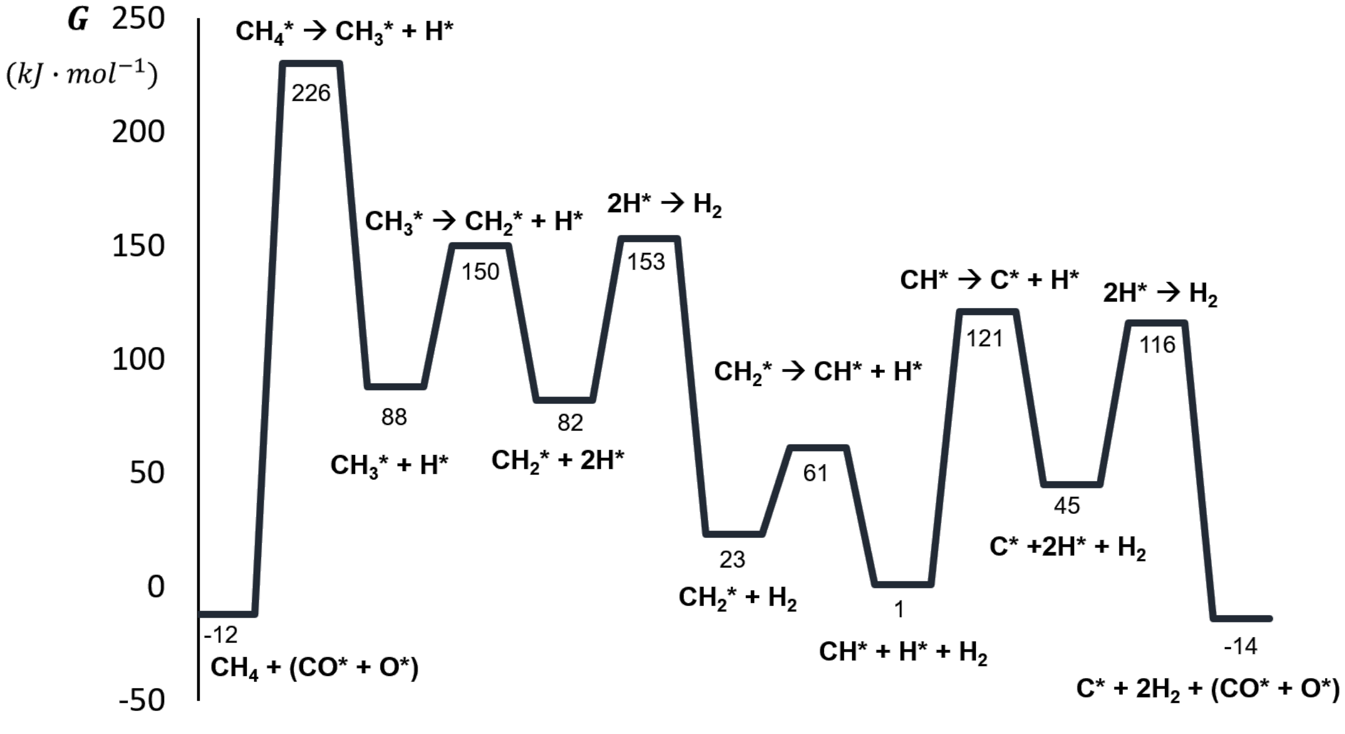

Supplementary Figure 25. Pathway to form C* and $2H_2$ from CH_4 involving the H–H coupling steps. Gibbs energies are given with respect to CH_4 and CO_2 (in kJ mol^{-1}).

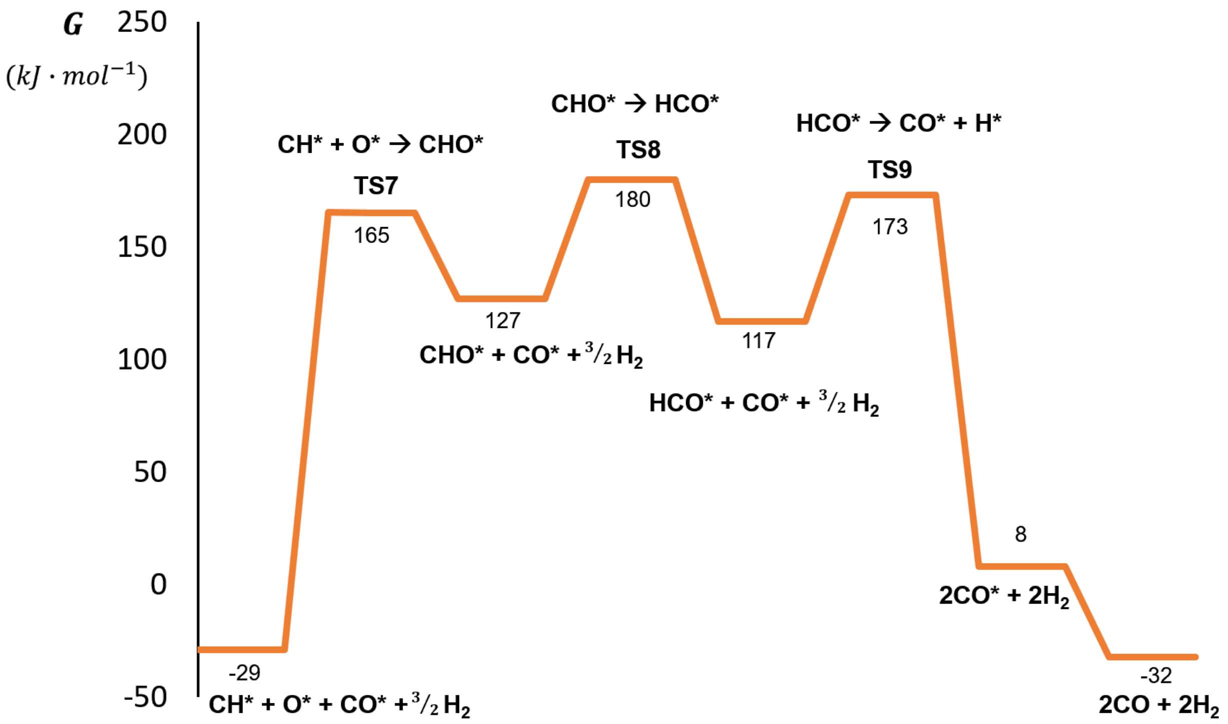

Supplementary Figure 26. Alternative oxidative pathway to form CO via the CHO* and HCO* intermediates. Gibbs energies are given with respect to CH_4 and CO_2 (in kJ mol^{-1}).

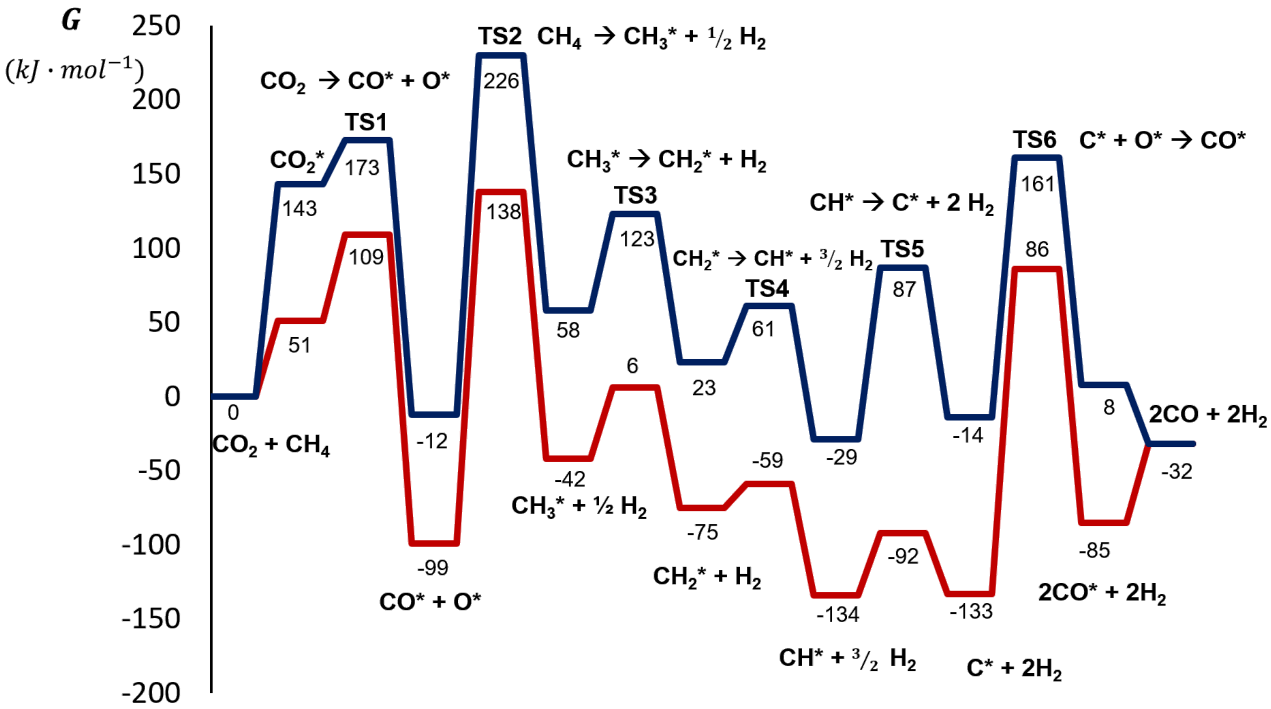

Supplementary Figure 27. Gibbs energy profile comparing the reactivities of 2D-Mo_2C-0.67 O ML (blue) and 2D-Mo_2C (red) surfaces.

Gibbs energies are given with respect to initial reactants, CH_4 and CO_2 (in kJ mol^{-1}). A star (*) notation indicates surface species, and related species are compared for 2D-Mo_2C-0.67 O ML and 2D-Mo_2C surfaces.

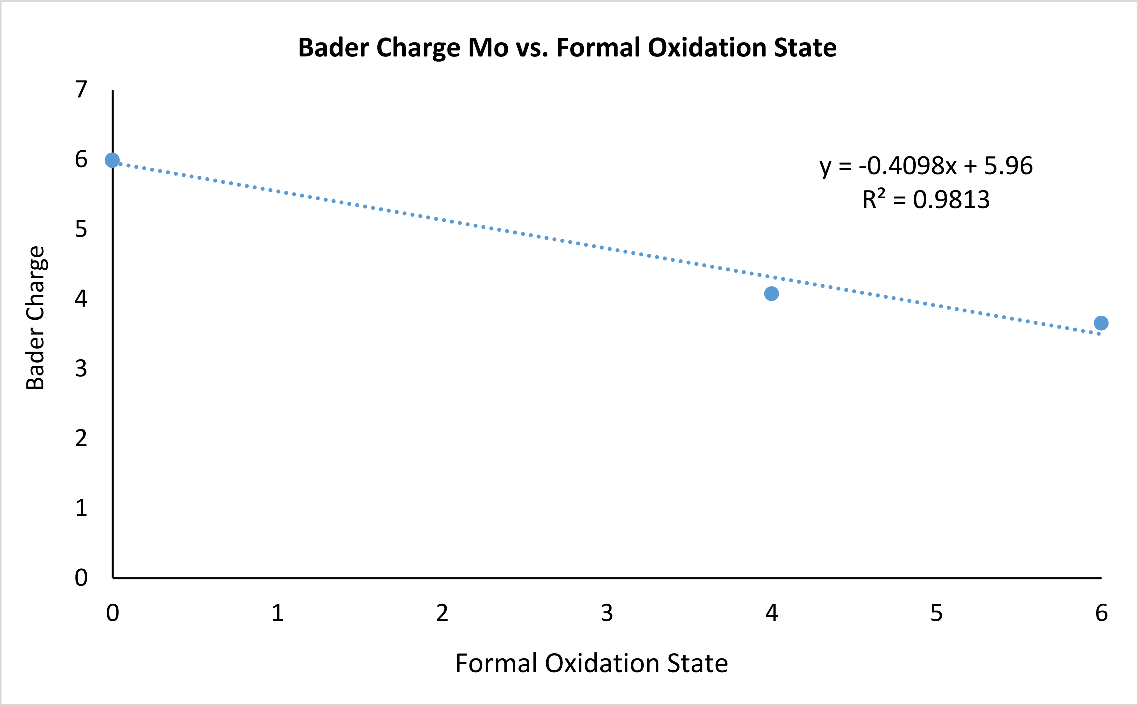

Supplementary Figure 28. Mo Bader charge dependence on the oxidation state (based on Mo, MoO_2 and MoO_3).

Supplementary Table 5. Oxidation states of the Mo atoms in the 2D-Mo_2C models with 1 O ML and 0.67 O ML oxygen coverage.

Oxidation State of Mo atoms (1.0 O ML)	Oxidation State of Mo atoms (0.67 O ML)
3.4	3.5
3.8	3.0
3.8	3.6
3.5	3.1
3.9	3.6
3.3	2.6
3.2	3.2
3.8	2.3
4.0	4.0
3.9	2.2
3.9	3.9
3.5	2.3
3.2	3.2
3.9	1.8
3.6	3.5
3.9	1.5
3.8	3.5
3.4	1.8

Supplementary Table 6. Comparison of the reactivity of 2D-Mo_2C-0.67 O ML with respect to 2D-Mo_2C surface (in kJ mol^{-1}). Energies are given with respect to the initial reactants, CH_4 and CO_2. A star next to the species indicates the related species is adsorbed either on the 2D-Mo_2C or 2D-Mo_2CO_x-surface (*).

Reaction Step	2D-Mo_2C-0.67 O ML G (kJ mol^{-1})	2D-Mo_2C G (kJ mol^{-1})
CO_2*	143	51
TS1 $CO_2 \rightarrow CO + O$	173	109
CO* + O*	−12	−99
TS2 $CH_4 \rightarrow CH_3* + H*$	226	138
$CH_3* + 1/2\ H_2$	58	−42
TS3 $CH_3* \rightarrow CH_2* + H*$	123	6
$CH_2* + H_2$	23	−75
TS4 $CH_2* \rightarrow CH* + H*$	61	−59
$CH* + 3/2\ H_2$	−29	−134
TS 5 CH* → C* + H*	87	−92
$C* + 2H_2$	−14	−133
TS6 C* + O* → CO*	161	86
$2CO* + 2\ H_2$	8	−85
$2CO + 2\ H_2$	−32	−32

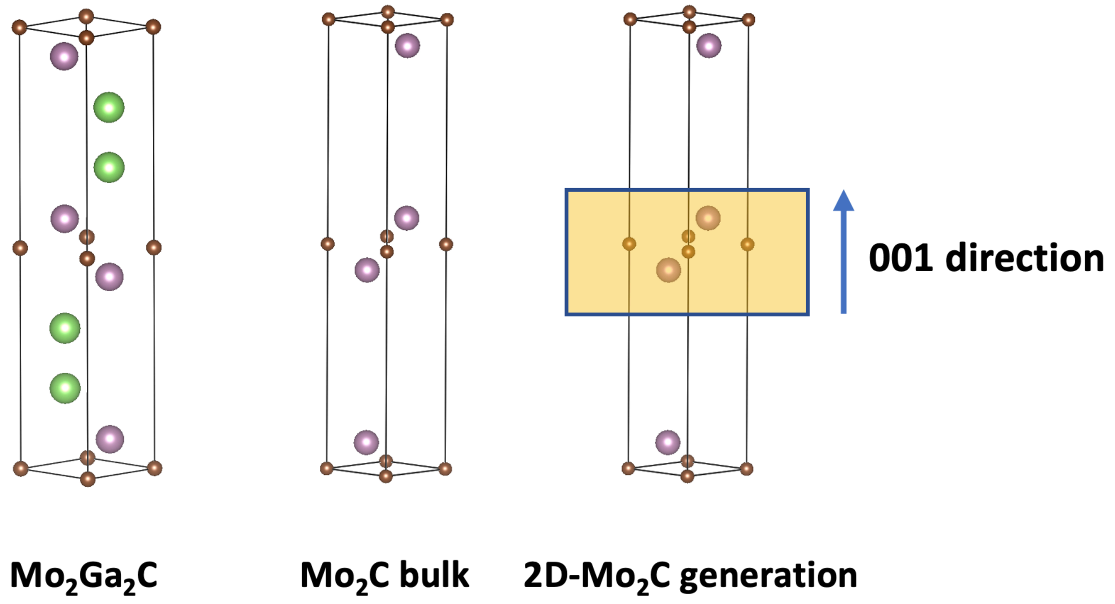

Supplementary Figure 29. Illustration of the construction of the theoretical model of the (001) facet of 2D-Mo_2C.

Supplementary References

1 Gao, H. *et al.* Simple and large-scale synthesis of β-phase molybdenum carbides as highly stable catalysts for dry reforming of methane. *Inorg. Chem. Front.* **5**, 90-99 (2018).

2 Yao, L., Wang, Y., Galvez, M. E., Hu, C. & Da Costa, P. Ni–Mo_2C supported on alumina as a substitute for Ni–Mo reduced catalysts supported on alumina material for dry reforming of methane. *C. R. Chimie* **21**, 247-252 (2018).

3 Yao, Z. *et al.* Insights into the deactivation mechanism of metal carbide catalysts for dry reforming of methane *via* comparison of nickel-modified molybdenum and tungsten carbides. *RSC Adv.* **6**, 19944-19951 (2016).

4 Zhang, S. *et al.* Catalytic role of β-Mo_2C in DRM catalysts that contain Ni and Mo. *Catal. Today* **258**, 676-683 (2015).

5 Zhang, A. *et al.* In-situ synthesis of nickel modified molybdenum carbide catalyst for dry reforming of methane. *Catal. Commun.* **12**, 803-807 (2011).

6 Liang, P. *et al.* Simple synthesis of ultrasmall β-Mo_2C and α-MoC_{1-x} nanoparticles and new insights into their catalytic mechanisms for dry reforming of methane. *Catal. Sci. Technol.* **7**, 3312-3324 (2017).

7 Brungs, A. J., York, A. P. E., Claridge, J. B., Marquez-Alvarez, C. & Green, M. L. H. Dry reforming of methane to synthesis gas over supported molybdenum carbide catalysts. *Catal Lett* **70**, 117-122 (2000).

8 York, A. P. E., Claridge, J. B., Brungs, A. J., Tsang, S. C. & Green, M. L. H. Molybdenum and tungsten carbides as catalysts for the conversion of methane to synthesis gas using stoichiometric feedstocks. *Chem. Commun.* **1**, 39-40 (1997).
